# Feasibility of a culturally adapted early childhood obesity prevention program among migrant mothers in Australia: a mixed methods evaluation

**DOI:** 10.1186/s12889-021-11226-5

**Published:** 2021-06-16

**Authors:** Sarah Marshall, Sarah Taki, Penny Love, Yvonne Laird, Marianne Kearney, Nancy Tam, Louise A. Baur, Chris Rissel, Li Ming Wen

**Affiliations:** 1 Sydney School of Public Health, University of Sydney, Camperdown, NSW 2006 Australia; 2 Health Promotion Unit, Population Health Research and Evaluation Hub, Sydney Local Health District, Forest Lodge, NSW 2037 Australia; 3 The National Health and Medical Research Council Centre for Research Excellence in the Early Prevention of Obesity in Childhood (EPOCH CRE), Sydney, Australia; 4 Institute for Physical Activity and Nutrition, School of Exercise and Nutrition Sciences, Deakin University, Waurn Ponds, Victoria 3216 Australia; 5 Sydney Medical School, University of Sydney, Camperdown, NSW 2006 Australia

**Keywords:** Prevention, Health promotion, Nutrition, Infant, Culture, Ethnicity, Implementation

## Abstract

**Introduction:**

Healthy Beginnings is an established nurse-led early childhood obesity prevention program that promotes healthy infant feeding practices and active play in the early years of life. To improve engagement with culturally and linguistically diverse populations, the Healthy Beginnings program delivered by telephone was culturally adapted and implemented with Arabic- and Chinese-speaking mothers in Sydney, Australia. The cultural adaptation process has been published separately. In this article, we aimed to evaluate the feasibility of the culturally adapted program.

**Methods:**

In 2018–2019, the culturally adapted Healthy Beginnings program was implemented with Arabic- and Chinese-speaking women recruited from antenatal clinics in Sydney. At four staged timepoints (from third trimester until 6 months of age), mothers were sent culturally adapted health promotion booklets and text messages and offered four support calls from bi-cultural child and family health nurses in Arabic and Chinese. A mixed methods evaluation included a) baseline and 6-month telephone surveys, followed by b) semi-structured follow-up interviews with a subset of participating mothers and program delivery staff. Main outcomes of this feasibility study were reach (recruitment and retention), intervention dose delivered (number of nurse support calls completed) and acceptability (appropriateness based on cognitive and emotional responses).

**Results:**

At recruitment, 176 mothers were eligible and consented to participate. Of 163 mothers who completed the baseline survey, 95% completed the program (*n* = 8 withdrew) and 83% completed the 6-month survey (*n* = 70 Arabic- and *n* = 65 Chinese-speaking mothers). Most mothers (*n* = 127, 78%) completed at least one nurse support call. The qualitative analysis of follow-up interviews with 42 mothers (22 Arabic- and 20 Chinese-speaking mothers) and 10 program delivery staff highlighted the perceived value of the program and the positive role of bi-cultural nurses and in-language resources. Mothers who completed more nurse support calls generally expressed greater acceptability.

**Conclusions:**

The culturally adapted Healthy Beginnings program was feasible to deliver and acceptable to Arabic- and Chinese-speaking mothers. Our results highlight the importance of in-language resources and individualised bi-cultural nurse support by telephone for supporting culturally and linguistically diverse migrant families with infant feeding and active play. These findings support the potential for program refinements and progression to an effectiveness trial.

**Supplementary Information:**

The online version contains supplementary material available at 10.1186/s12889-021-11226-5.

## Background

Women from culturally and linguistically diverse backgrounds who migrate to high-income English-speaking countries, such as Australia, can face challenges accessing child and family health services. This is partly due to language barriers, unfamiliarity with a different health system and cultural differences [1–3]. With migration increasing worldwide [4], reducing health inequities among culturally and linguistically diverse migrants is a global priority.

In Australia, women and infants from culturally and linguistically diverse backgrounds, compared to Australian-born women, are less likely to use health services [5], more likely to report negative experiences of maternity care [6] and more likely to report depressive symptoms [7]. Reduced access to healthcare impacts the support mothers receive for their health and the health of their children in this critical early period of life. Yet, there is a lack of evaluated interventions to improve access to postnatal care for culturally and linguistically diverse migrant families [8].

Infant feeding practices and active play are among the known early-life modifiable factors for healthy weight gain and the prevention of obesity [9–12]. A recent meta-analysis found that migrant women are more likely to start and maintain any breastfeeding, and can face challenges to exclusively breastfeed [13]. Migrant women experience disruptions to usual practical and emotional supports which can negatively impact infant feeding practices [14, 15].

In Australia, children from culturally and linguistically diverse backgrounds experience higher prevalence rates of overweight and obesity when compared with English-speaking Australian-born children [16]. Early interventions targeting individual-level behaviours of parents of infants are one approach for promoting healthy early-life behaviours and preventing obesity [17]. Randomised controlled trials (RCT) of such programs in Australia have yet to focus on cultural minority populations [18–21]. To achieve health equity, healthcare and programs supporting early healthy growth must be culturally relevant and accessible to women and children from culturally and linguistically diverse backgrounds.

### Cultural adaptation of the Healthy Beginnings program

Healthy Beginnings is a child and family health nurse-led early childhood obesity prevention program providing individual support for mothers and their infants [18]. At staged timepoints starting antenatally, mothers receive support focussed on infant nutrition, active play and sedentary behaviours. The first Healthy Beginnings RCT, with nurse home visits, showed reduced mean body mass index and improved feeding practices among children in the intervention group at age 2 years [22, 23]. The behavioural outcomes were also found in a subsequent RCT (Communicating Healthy Beginnings Advice by Telephone [CHAT]) with nurse support calls or Short Message Service (SMS) support [24, 25], however outcomes were poorer for mothers who spoke a language other than English at home [26].

**Fig. 1:**
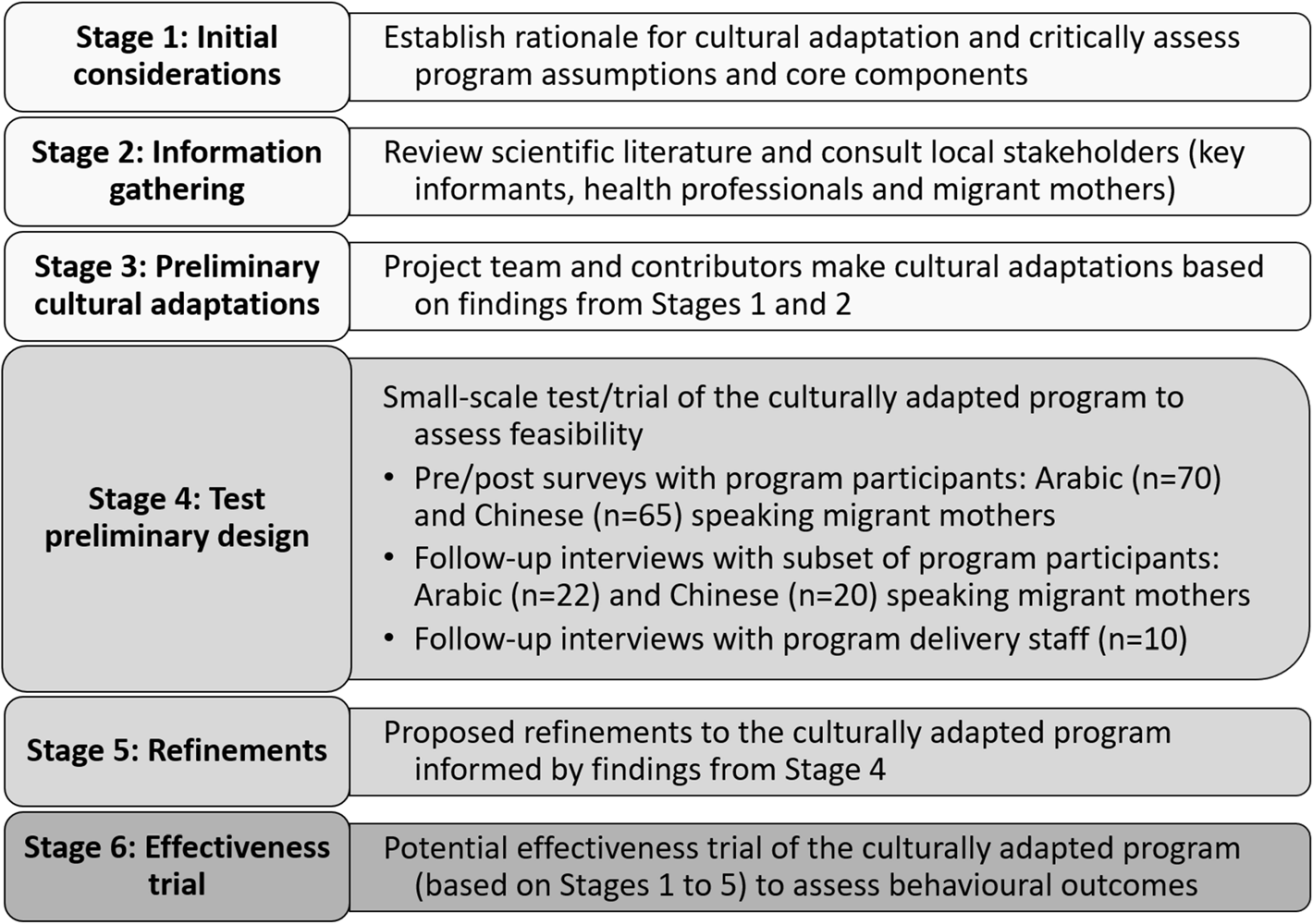
Overview of the stages for cultural adaptation of Healthy Beginnings. Stages 1–3 are reported separately [29]

In 2018–2019, we undertook a project to culturally adapt the Healthy Beginnings telephone-delivered program (CHAT), herein referred to as the culturally adapted Healthy Beginnings. Arabic and Chinese language groups were the focus for the adaptation as these languages are most commonly spoken after English in central Sydney [27]. The cultural adaptation process was based on Barrera and colleagues’ adaptation model [28]; the stages are presented in Fig. 1. Stages 1–3 have been reported separately [29] and are briefly described here.

The first stage included assessing the underpinning theories and core components of the Healthy Beginnings program. These core components included: a) key focus areas related to infant feeding, active play, sedentary behaviours and sleep; b) behaviour change techniques; and c) delivery mode. The second stage included reviewing scientific literature and consulting with Arabic- and Chinese-speaking mothers, as well as child and family health professionals. The findings highlighted the importance of family, bi-cultural doctors and culturally sensitive health services for supporting infant feeding [30], as well as common practices and beliefs such as breastfeeding as part of the social norm among Arabic-speaking mothers, and concerns about perceived low breastmilk supply among Chinese-speaking mothers [29]. The third stage included making cultural adaptations to Healthy Beginnings based on the findings of Stages 1 and 2. The key adaptations included recruitment approaches, staffing (bi-cultural nurses and project staff) and program content (culturally adapted and translated written health promotion booklets and SMS).

### Aims of the current study

Feasibility studies are essential for exploring relevance and acceptability of complex interventions [31], particularly in adaptation [32] and cultural adaptation studies [28] where the intervention is applied to a new context. Our overarching aim was to investigate the feasibility of delivering the culturally adapted Healthy Beginnings program among Arabic and Chinese-speaking migrant mothers. Our primary objectives were to determine:

- reach among the intended population;
- engagement in the nurse support calls; and
- acceptability among participants and delivery staff.

These findings will guide program refinements (Stage 5, Fig. 1) and inform the appropriateness of an effectiveness trial (Stage 6, Fig. 1) to understand the culturally adapted program’s impact on key infant behaviours.

## Methods

### Study design

This study used a single-arm/non-randomised sequential mixed methods design to investigate the feasibility of the culturally adapted Healthy Beginnings program delivered by telephone [29]. This design suited well for testing the newly adapted program and proposing refinements, representing Stages 4 and 5 of the cultural adaptation process (refer to Fig. 1). We undertook this study in 2018–2019 with Arabic- and Chinese-speaking women in Sydney, Australia from their third trimester of pregnancy until their baby was aged 6 months. Bi-cultural research staff administered telephone surveys with participating mothers at baseline and 6-months. All participating mothers and program delivery staff were invited to participate in a follow-up interview after the program.

Administrative and survey data were used to describe participant recruitment, retention and engagement with nurse support calls. Interviews with a subset of the participants and program delivery staff were used to explore acceptability.

The Ethics Review Committee of the Sydney Local Health District granted ethical approval of this study (protocol X18–0049, reference HREC/18/RPAH/82).

We followed CONSORT reporting guidelines for reporting of this non-randomised feasibility study (see Additional file 1) [33, 34].

### Program implementation

#### Context

In Australia, all women and infants are eligible to access free universal maternity and child and family health services, however services differ across Australian states and territories [35]. During pregnancy, appointments with nurses and midwives are offered through public hospitals. After birth, there is a transition to postnatal care delivered by child and family health nurses (CFHN) in community health centres. In Sydney, New South Wales, language-specific services can be dependent on local community needs and staff availability.

#### Program design

The program and cultural adaptations are outlined in the Background section and reported in detail separately [29]. Four nurse support calls, offered in either Arabic or Chinese (Mandarin), were staged according to feeding and active play program messages and infant age (antenatal during the third trimester of pregnancy, then at infant age 1, 3 and 5 months). The nurse calls followed the program scripts with prompts. Participants were also sent corresponding culturally adapted and translated program booklets in the mail and SMS messages in Arabic or Chinese. The SMS system was two-way, allowing mothers to reply with queries for the research team and/or nurses to respond. The culturally adapted booklets are available to download from the project website [36].

#### Program delivery

Bi-cultural CFHNs were available 1 day per week and made four call attempts to mothers at each time point. If mothers phoned or sent an SMS message requesting urgent advice when the bi-cultural CFHN was not available, then mothers were offered the option to speak with an English-speaking CFHN and an interpreter and also referred to their family doctor. Mothers who could not be reached after four call attempts for the first (antenatal) nurse call were moved to a ‘no-calls’ group but continued to receive the program booklets and two-way SMS messages.

### Participants

#### Eligibility

Women were eligible if they were aged at least 18 years, 28–36 weeks pregnant, owned a mobile phone and were able to speak and read Arabic or Chinese-Mandarin. Exclusion criteria included having a self-reported medical condition that might affect feeding plans or a known foetal abnormality. All program delivery staff were eligible for follow-up interviews.

#### Recruitment

From August until December in 2018, eligible women who attended antenatal clinics in three local health districts in Sydney were invited to participate by bi-cultural research staff or English-speaking research staff with professional medical translators. All participants were informed about the research and provided translated study information and consent forms. Eligible and consenting women completed a registration form with research staff and arranged a suitable time to complete the baseline telephone survey. Mothers were provided a $20AUD grocery voucher for each telephone survey completed (baseline and 6-months) and for participating in an interview.

Program delivery staff directly involved with participants during the program (recruitment, surveys, nurse calls) (*n* = 12) were emailed an invitation to participate in a brief demographic survey and an interview to discuss program adaptation and delivery. Voluntary participation was emphasised. Staff did not receive compensation.

#### Sample size

We aimed to recruit 70 mothers from each language group. The sample size was determined by project resources, seeking diversity of participant demographics and geographical location (factors that may affect engagement), and also allowing for anticipated attrition of approximately 20%, based on the Healthy Beginnings trial [18]. As the aim was not to evaluate effectiveness, a power calculation for sample size was not applicable [37].

Follow-up interviews were conducted with a nested sample of approximately 20 mothers from each language group. This target was based on project resources and availability of interpreters. The sampling was purposeful; quantitative survey results and administrative data were used to seek a mixed sample of mothers who: a) were first-time mothers or had more than one child (multiparous); b) completed 0–1 of the telephone support nurse calls (lower intervention dose) or 2–4 of the nurse calls (higher intervention dose); c) were living in Australia for less time (0–5 years) or more time (6+ years). All staff who consented and were available were interviewed.

### Data collection

#### Administrative data

Administrative data, such as participation in nurse calls, were recorded by bi-cultural CFHNs and project staff. Mothers who withdrew from the study were invited to provide a reason, which was recorded. All data capture and management were via REDCap (Research Electronic Data Capture) for conducting online surveys, hosted at Sydney Local Health District, Australia [38].

#### Quantitative baseline and 6-month surveys with mothers

Mothers participating in the program were invited to complete a telephone survey (approximately 20–30 min) at baseline (28–36 weeks gestation) and at the end of the program (infant age 6 months). The survey questions were based on previous Healthy Beginnings research protocols [18, 24] and included mothers’ socio-demographic characteristics, program satisfaction, mothers’ physical activity and diet, perceptions and practices related to key infant behaviours. See Additional file 2 for the surveys in English.

Surveys in English were translated into Arabic and Simplified Chinese by professionals accredited by the Australian National Accreditation Authority for Translators and Interpreters. The translated surveys were tested for face validity with two Arabic- and Chinese- speaking mothers of young children not participating in the study, and bi-lingual project team members made refinements to ensure clarity of meaning. The process was guided by survey translation and adaptation methods [39]. Trained bi-cultural research staff conducted the surveys in language over the phone and entered responses into REDCap in English.

#### Qualitative follow-up interviews with mothers and staff

- Mothers who participated in the program

During the 6-month survey, mothers were invited to participate in a follow-up telephone interview in Arabic or Chinese to talk about their experiences being part of the program. A purposive sample was invited to interview. The interviews followed a semi-structured guide with open-ended questions about mothers’ views of the program generally; their thoughts about program booklets, SMS messages and nurse calls; and behaviour changes related to the program (see Additional file 3). The interview guide was pilot tested with one program participant, and minor refinements were made to wording and sequence of the interview questions. This interview was included in the analysis.

Interviews were co-facilitated by English-speaking staff members, SM or MK, with bi-lingual staff member NT or one of two professional Arabic interpreters from Sydney Local Health District Interpreter Service. SM, MK and NT are all experienced health promotion practitioners with research training. Co-facilitators were briefed about the study and the interview guide. The role of the interpreter as co-facilitator is a recognised approach for engaging participants in cross-cultural research [40]. The co-facilitating interpreter asked the questions in Arabic or Chinese and provided a summary for SM/MK in English, who then directed subsequent questions. Interviews were audio-recorded with participant consent. Facilitators debriefed after each interview and SM/MK wrote reflective notes. Interviews averaged 37 min (range 20 to 60 min). The sections of the audio recordings in English were transcribed and checked by NT and professional transcribers.

b) Program delivery staff

For staff who were available and consented, interviews were over the telephone or in-person at their workplace. A semi-structured guide was used to ask open-ended questions about their experience generally; their thoughts about the program booklets, SMS messages and nurse calls; and project implementation (see Additional file 4). The guide was pilot tested once by SM with a staff member who was familiar but not involved with Healthy Beginnings (results excluded) and SM subsequently refined the wording and sequence of the interview questions.

Interviews were facilitated by SM in English. Staff were known to SM only in the context of this project. SM is a PhD candidate with public health experience and research training. Interviews were audio-recorded with participant consent. Notes were taken after all interviews to document reflections and to inform subsequent interviews. Interviews averaged 54 min (range 25 to 90 min). Interviews were professionally transcribed, and SM checked all transcripts for accuracy.

### Outcome measures

The outcomes investigated in this cultural adaptation feasibility study are outlined in Table 1 and described below. These outcomes align with guidance for conducting non-randomised feasibility studies [41] as well as the Stages of Cultural Adaptation model [28] used to guide this cultural adaptation.

**Table 1:** Summary of feasibility outcome measures

Outcomes	Research questions	Data sources and measures
Reach	Were the intended target populations recruited? Were participants retained?	Administrative data: Number of mothers who enrolled and completed the surveys Baseline survey data (mothers): Demographics of mothers
Intervention dose delivered	To what extent did recruited participants engage in the intervention? What was the quantity of the intervention received/completed?	Administrative data: Number of nurse support calls completed
Acceptability	To what extent was the program (delivery and content) perceived as acceptable to participating mothers and program delivery staff? How satisfied were mothers with their overall experience of the program?	Follow-up interview data (mothers and program delivery staff)

#### Reach and intervention dose delivered

Reach and dose delivered were assessed using administrative and survey data including participant enrolments, demographics, nurse calls completed and retention at the 6-month survey. The demographic variables included age, parity, country of birth, years in Australia, marital status, education, income and religion.

#### Acceptability

We defined acceptability according to the Theoretical Framework of Acceptability (TFA) [42], where acceptability is considered to be “the extent an intervention is considered appropriate based on cognitive and emotional responses”. Acceptability of the culturally adapted Healthy Beginnings program was primarily explored retrospectively through qualitative interviews with a subset of participating mothers and program delivery staff. Additionally, at the 6-month survey we asked mothers about their overall satisfaction with the program to gain a quantitative assessment from all participants and to assist with prompting discussion in the interviews. We used the eight-item Client Satisfaction Questionnaire (CSQ-8) [43] (findings presented in Additional file 5) and an open ended question inviting comments about the program.

### Data analysis

The quantitative and qualitative data were equally weighted, linked between data collection points and mixed at data interpretation stages. The goal of mixing quantitative and qualitative methods was to enhance our understanding and interpretation of the feasibility of the culturally adapted program. We took a pragmatic paradigmatic lens when analysing and interpreting the data, emphasising social experience to prioritise actions [44].

Quantitative data was transferred from REDCap to IBM SPSS Statistics [45]. Descriptive statistics were used to report sociodemographic characteristics, nurse support calls completed, satisfaction scores and retention. Chi-square tests were conducted to investigate the differences in sociodemographic characteristics between mothers who completed the baseline and 6-month surveys.

Qualitative data were analysed using descriptive thematic analysis following the Framework Method [46]. SM analysed the interviews using the Theoretical Framework of Acceptability (TFA) [42] constructs (described in Table 3) as overarching categories and developed codes based on the data. This use of deductive and inductive coding, connecting TFA theory and the data, is consistent with pragmatic lens of abductive reasoning [47]. SM read and listened to all interviews, then coded one interview from the Arabic mothers, Chinese mothers, and staff to develop an initial coding framework. Co-authors ST, YL, MK, NT, YL also independently coded these first three transcripts using the TFA categories to generate ideas and develop the initial coding framework.

Transcripts were then imported into NVivo 12 for data management. SM continued to code the data, starting with the mothers' interviews, and refining the coding framework until the final staff interview was coded. SM then charted the data, from mothers and staff separately, into matrices for each TFA construct, applying further codes as required. For coding consistency, initial transcripts were revisited for additional coding. SM made notes to interpret the data, continually comparing findings for the mothers based on number of children, nurse calls completed and years in Australia. Our intention with these comparisons was to explore any differences, as we hypothesised experiences and opinions of the program may have been more varied. SM then developed themes within the TFA constructs to represent shared meaning across participants. Co-author group meetings were held throughout this process for generating ideas, discussion and refining themes. The co-author group agreed upon the final themes presented.

## Results

### Program participant recruitment and flow

**Fig. 2:**
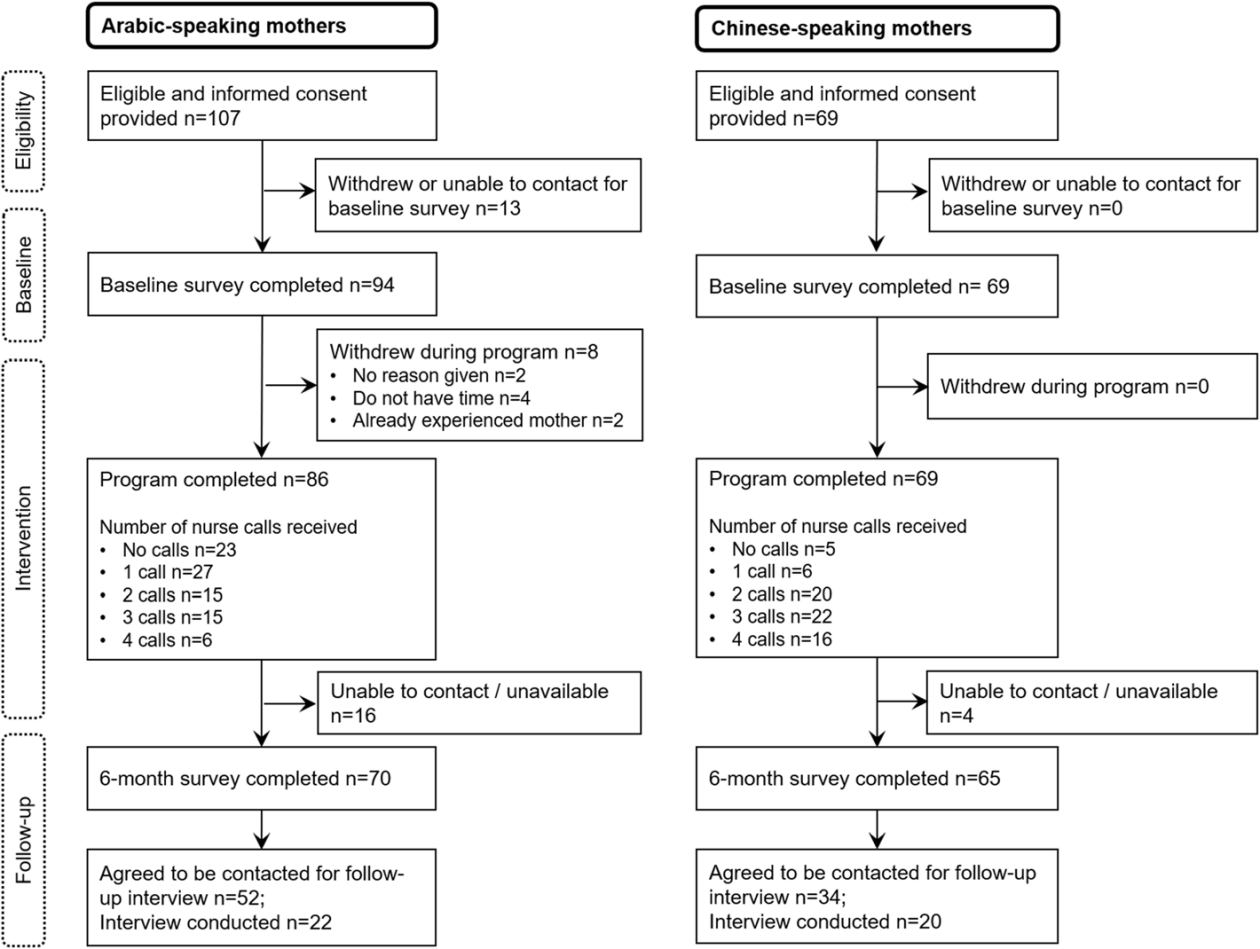
Flow diagram of program participants

Mothers’ participation in the study is presented in Fig. 2. Of 176 eligible and consenting mothers, 163 enrolled and completed the baseline survey (*n* = 94 Arabic- and *n* = 69 Chinese-speaking mothers). Eight mothers withdrew during the program. Most mothers (*n* = 127, 78%) completed at least one nurse call, and one third (*n* = 59, 38%) completed at least three of the four calls. A small group of mothers (*n* = 23 Arabic- and *n* = 5 Chinese-speaking mothers) could not be reached for the first nurse call after four attempts and were moved to a ‘no-calls’ group. Of mothers who enrolled, 135 completed the 6-month survey (at infant mean age 5.6 months, range 4.3–7.9 months); a retention rate of 83%.

### Demographic characteristics of program participants

Demographic characteristics of the mothers who completed the baseline and 6-month surveys are presented in Table 2. Arabic-speaking mothers were born in 12 different countries (Iraq *n* = 30; Lebanon *n* = 29; Egypt *n* = 7; Syria *n* = 6; Iran, Jordan, Sudan *n* = 2; Kuwait, Libya, Saudi Arabia, Morocco *n* = 1). Chinese-speaking mothers were born in four different countries or regions (China *n* = 65; Taiwan *n* = 2; Hong Kong, Vietnam *n* = 1).

**Table 2:** Demographic characteristics of mothers participating in the program at baseline

Variables, n (%)	Arabic-speaking mothers (*n* = 94)	Chinese-speaking mothers (*n* = 69)
**Mothers’ age**		
18–24	13 (14)	4 (6)
25–34	53 (56)	46 (67)
35–44	28 (30)	19 (28)
**First-time mother**		
Yes	22 (23)	45 (65)
No	65 (69)	23 (33)
**Years in Australia**		
< 6 years	36 (38)	31 (45)
≥ 6 years	45 (48)	37 (54)
**Annual household income**		
< $ 80,000 AUD	12 (13)	22 (32)
≥ $ 80,000 AUD	4 (4)	32 (46)
Do not know/prefer not to answer	78 (83)	15 (22)
**Employment status**		
Employed (full or part-time/casual)	7 (7)	33 (48)
Unemployed, home duties, other	87 (93)	36 (52)
**Education level**		
Up to secondary school	51 (54)	7 (10)
Technical college/diploma	20 (21)	9 (13)
University degree or higher	23 (24)	53 (77)
**Religion**		
No religion	0 (0)	58 (84)
Buddhism	0 (0)	6 (9)
Christianity	18 (19)	5 (7)
Islam	58 (62)	0 (0)
Mandaeism	16 (17)	0 (0)

Note: Values do not always equal 100% due to missing values

*p < 0.05; Analysis by Chi-square test; *AUD* Australian Dollars

The demographic characteristics of the Arabic-speaking mothers who completed the baseline (*n* = 94) and the 6-month (*n* = 70) surveys showed significant differences in their years in Australia (*p* = 0.036) and religion (*p* = 0.018). There were no significant differences in demographic characteristics of the Chinese-speaking mothers who completed baseline (*n* = 69) and 6-month (*n* = 65) surveys.

### Acceptability of the program

#### Characteristics of interview participants (mothers and program delivery staff)

At program completion, interviews were conducted with program participants (*n* = 22 Arabic- and *n* = 20 Chinese-speaking mothers) and with program delivery staff (*n* = 10). Additional file 6 presents demographic characteristics of mothers who agreed to be contacted, and those who completed an interview. Three mothers who consented to the interview did not agree to the audio recording, and in these cases detailed notes were taken.

Approximately half of the Arabic-speaking mothers interviewed were newer arrivals (< 6 years in Australia) (*n* = 12) and had completed either one nurse call (*n* = 10) or two or more calls (*n* = 12). Few were first-time mothers (*n* = 4). Approximately half of the Chinese-speaking mothers interviewed were newer arrivals (< 6 years in Australia) (*n* = 10) and first-time mothers (*n* = 10). Most had completed two or more calls (*n* = 19). While we sought greater representation of Arabic-speaking mothers who were first-time mothers and Chinese-speaking mothers who completed fewer calls, however not all mothers agreed to the interview nor could be contacted, and our interview sample characteristics were reflective of the total sample.

Program delivery staff who participated in interviews were involved with the cultural adaptations, recruitment, survey administration and/or nurse calls. Half were CFHNs (*n* = 5), and half were project officers/assistants (*n* = 5). Staff had an average of 5 years of experience in a similar role (range 1–11 years). Most were fluent in languages other than English (Arabic *n* = 4 or Chinese *n* = 4).

#### Thematic analysis findings

Using the Theoretical Framework of Acceptability (TFA) constructs, we developed themes based on the data to represent shared meaning (Table 3). Generally, mothers and program delivery staff who were interviewed felt that the culturally adapted Healthy Beginnings program was unique and valuable. Almost all mothers said they would recommend the program to others. The thematic analysis findings are described below. Quotes from mothers are presented with their language group, whether they were first-time mothers or had more than one child (first-time/multiparous), and their years living in Australia. Quotes from staff are presented with their role and language(s) spoken. All quotes include an interview identification (ID) number.

**Table 3:** Theoretical Framework of Acceptability (TFA) constructs and thematic analysis findings from follow-up interviews with mothers (*n* = 42) and program delivery staff (*n* = 10)

TFA construct and definition	Themes derived
Ethicality: The extent to which the intervention fits with an individual’s value system	• Value of language and cultural understanding • Written booklets as a tool for involving family and friends • Healthy Beginnings as a trustworthy program
Affective attitude: How an individual feels about the intervention	• Mothers generally liked the combination of delivery modes • Mothers appreciated the time and emotional support from bi-cultural nurses • Age-appropriate information helped mothers with what to expect
Perceived effectiveness: The extent to which the intervention is perceived to have achieved its intended purpose	• The bicultural nurse gave specialised advice and helped mothers with problem solving • The program SMS and booklets for increasing knowledge and reminders • Perceptions that the program was especially useful for first-time mothers and new migrants
Burden: The effort needed to participate	• Logistical issues increased mothers’ efforts needed to participate • The phone-based service was seen as convenient • Mothers are busy and did not always have time
Intervention coherence: The extent to which the intervention is understood	• Uncertainties about the program impacted involvement

The TFA has seven constructs and after inductively coding the data using these constructs as overarching categories, we found that five were represented with our data (see Table 3). The two TFA constructs that were not strongly represented were: ‘Opportunity costs: the extent to which benefits, profits, or values must be given up to engage in the intervention’ and ‘Self-efficacy: the participant’s confidence that they can perform the behaviour(s) required to participate in the intervention.’ Mothers consented to participate in this feasibility study, so this does give an indication of their confidence to participate (i.e., confidence to receive booklets, SMS messages and nurse calls), and factors that may influence this (such as understanding the program and being able to schedule with the nurse) are described within the other presented constructs. Self-efficacy to undertake target behaviours of the program clustered with the TFA construct ‘perceived effectiveness’.

### Construct 1: Ethicality

#### Value of language and cultural understanding

Mothers commonly identified ease and preference for using their first language, regardless of how many years they had lived in Australia and their English language proficiency: “*It is wonderful to talk to someone using your own language. English is still ok, but it is a second language and not as good as mother language. […] I feel I can ask more questions that I want, compared with other nurses in English”* (ID8; Chinese; multiparous; 11 years). Some mothers explicitly spoke of the importance of cultural congruence with the nurse: *“It was easier and flowing because also we’d talk about culture or traditions, […] so we were able to discuss any topic possible in regard to health and culture”* (ID18; Arabic; multiparous; 11 years). Staff endorsed the value of the program, emphasising the importance of cultural support offered to mothers: “*Because I understand her. Because with more understanding you actually give more empathy”* (ID1; CHFN; Mandarin, English).

#### Program booklets as a tool for involving family and friends

Mothers described the program booklets as useful for sharing the program information and discussing new ideas with fathers, family members and friends: *“The booklet for me is the evidence for me to show that this is correct. I find it helpful”* (ID7; Chinese; first-time; 1 year). Some mothers, particularly those who had been in Australia for longer, talked confidently of using the booklets as a tool for advocating for new behaviours with family members: *“When I gave my mother the booklets to read, she got convinced and said this is your era and we are from a different era [laughs]. So now she understands why you feed the way you do”* (ID9; Arabic; first-time; 9 years). This was especially relevant when family members had differing views, for example, introducing solids around 6-months and tummy-time practices: “*So mainly my in-laws and my mother, they don’t support the tummy-time ideas. They find [that] the baby’s too small, you shouldn’t do that”* (ID18; Chinese; multiparous; 5 years). Staff interview data did not represent this theme.

#### Healthy Beginnings as a trustworthy program

Mothers regarded the culturally adapted Healthy Beginnings program as trustworthy and credible, being developed and delivered by the government health service and health professionals: *“Because I knew nothing and these people who wrote the information, they have the experience”* (ID102; Arabic; first-time; 0.5 years). Additionally, some mothers referenced the value of evidence-based information: “*During my pregnancy, I have seen in Australia a lot of research on how to raise a baby […] the research can be trusted. Like in China, it is just like old experience, I think*” (ID19; Chinese; first-time; 9 years). Staff interviews supported this view: *“They believe that health departments provide the correct, most updated information”* (CFHN; Mandarin, Cantonese, English); especially the perceived acceptability of the program being delivered by a nurse: *“A nurse is a very acceptable profession by many cultures”* (CFHN; English). Staff also found that trustworthiness was important for recruitment: *“I would introduce myself and show my New South Wales Health ID just to trust me”* (Project assistant; Arabic, English).

### Construct 2: Affective attitude

#### Mothers generally liked the combination of delivery modes (SMS, booklets, nurse calls)

Many mothers described the suite of offerings from Healthy Beginnings as suiting their needs and they liked that the content was consistent across the nurse calls, booklets, and SMS messages: *“At first, every bit of information is helpful, from the three different types of support. Because the book is comprehensive, a bit of the tailor-made answers from the nurses, even the SMS is similar to the apps, you get some other extra bits”* (ID16; Chinese; first-time; 2.5 years). For mothers who received calls, this was overwhelmingly the preferred element of the program: “*It was very useful. I preferred to have the telephone calls, because I am able to ask questions”* (ID19; Arabic; first-time; 5 years). Many mothers and staff appreciated the paper-based resources, but there were several suggestions to add a website or app so that mothers could easily search for relevant program information online. Few suggested other modes such as social media groups (e.g., WeChat or Facebook) or face-to-face groups, however opinions differed about the uptake of these formats.

#### Mothers appreciated the time and emotional support from bi-cultural nurses

Many mothers expressed appreciation that the nurse gave time and patience to address their specific questions, and all mothers valued having the calls available in their language. The bi-cultural CFHN was important for providing emotional support for mothers: *“She spoke to me a few times, I was very moved. The nurse initiated the call and picked up in my voice; she noticed that I must be tired. I was the only one looking after the baby. [The nurse] asked whether there’s any issues and initiated help”* (ID10; Chinese; multiparous; 5 years). This was especially relevant when family supports were not present: *“In my case, I don’t have any relatives around me and very limited friends. So, yeah, it was nice to have that call from [Healthy Beginnings nurse] that time”* (ID11; Arabic; multiparous; 10 years).

Compared to experiences with the doctor or nurses at Community Health Centres, the bi-cultural CFHN discussed maternal as well as infant health and could give more time to building rapport and addressing mothers’ concerns: *“I find that [the nurse’s] help is very good, because it is helpful to talk with someone, and for her to give me a call and spend quite good time with me. This conversation gave me some comfort”* (ID20; Chinese; first-time; 15 years). The CFHNs commented that they felt their calls were especially helpful for first-time mothers with many questions, and earlier in the program when mothers were more in-need and motivated. They also felt that more nurse availability could increase their support: “*I think maybe because I’m speaking their language, it’s the first, sort of people they want to talk to. So, I would actually, I know it’s probably a luxury, but recommend to have a full-time nurse and be able to do these things full-time. That would be wonderful. That will be providing more service for them”* (CFHN; Chinese, English).

#### Age-appropriate health information helped mothers with what to expect

Generally, mothers felt the timing of information was appropriate to their baby’s development: *“I learned about the baby’s stages of their growth and development each month, and what sort of skills they acquire, how to deal with them, and how to feed them”* (ID21; Arabic; multiparous; 0.5 years). Anticipatory information seemed to reduce stress for some mothers, irrespective of having previous children or number of years in Australia: *“The information is helpful to prepare with what to expect. When things happen, I wasn’t as concerned and not so anxious”* (ID9; Chinese; multiparous; 6 years). Staff supported this view: *“The information is quite age appropriate, that’s what I feel. Like, this is basically what we need to transfer to the parent in that specific period”* (CFHN; Chinese, English).

### Construct 3: Perceived effectiveness

#### The bicultural nurse gave specialised advice and helped mothers with problem solving

Most mothers who completed nurse calls talked positively about their experiences with the bi-cultural CFHN – describing the nurse as helpful, providing new information and strategies to help overcome problems. The most common examples were related to breastfeeding challenges such as milk supply, latching, damaged nipples and expressing milk. Other examples included managing shared care with family, strategies for settling and resolving problems with tummy-time positions. One mother described her experience with breastfeeding difficulties: *“I tried the way they teach me, and my milk increased a lot. Still, I had to use formula as well as breastfeeding the baby. But my milk eventually increased a lot more compared to the beginning. I’m really happy about it. I didn’t breastfeed my first baby but this time, I had help from booklets and phone call. I have built up a lot more milk compared to last time with my daughter”* (ID8; Chinese; multiparous; 11 years).

Some mothers who received none or one nurse call felt less supported. One mother, who only received the antenatal call, described an extreme case where she was not able to find help: “*My baby was about five weeks and she stopped feeding completely. She just turned off, you know. She didn’t want to take the breast at all […] I didn’t know what was happening, and I didn’t know where to go to*” (ID3; Arabic; multiparous; 17 years). This ties in with a reflection of one of the bi-cultural CFHNs about needing to consider additional supports, such as online resources, for mothers to also seek their own answers too: *“Rather than relying on the nurse or relying on the booklet, we also want them to be proactive to be able to look for the information driven by their needs […] I think that’s the skill as a healthcare professional we should promote”* (CHFN; Mandarin, English). This links with suggestions to include program advice online (see Construct 2).

Almost all mothers visited their family doctor, who was often bi-cultural, for medical advice, immunisations and physical checks. Arabic- and Chinese-speaking mothers referred to the importance of the doctor’s role, particularly for urgent assistance: *“For more immediate medical issues or problem then the first thing I think of is to go to the doctor”* (ID17; Chinese; multiparous; 11 years). Yet, when making a direct comparison with the bi-cultural CFHN, mothers often referred to them as having more time, specialised advice and support: *“the family doctor will give me medicine, but [the Healthy Beginnings nurse] was different. She made me feel mentally comfortable”* (ID14; Arabic; multiparous; 3 years). Few mothers attended Community Health Centres (CHC), but those that did said that the CHC face-to-face visits were useful for demonstrations and growth measurements, but that the consultations were more generalised and time-limited. Mothers who did not attend their CHC talked about barriers such as being busy, not knowing the location, uncertainty of nurses’ language, and difficulties using medical terms: “*With the nurse outside [at CHCs], although you speak English, can have simple English conservation, sometimes the medical terms are not easy, you also have to go through a lot of travelling time, and pre-book an appointment”* (ID11; Chinese; first-time; 2 years). Compared to the doctor and the CHC service, mothers highlighted that the bi-cultural CFHN nurses offered convenience over the phone (relates to Construct 4) and specialised parenting advice. In terms of advice and problem solving, the bi-cultural CFHNs made reference to doctors’ advice and were particularly concerned about mixed messages: *“A good portion of the mums have said their GP [general practitioner/ family doctor] has said to start solids now at 4 months*” (CFHN; Arabic, English). For staff, this emphasised the need for mothers to receive the nurse calls and consistent messaging, acknowledging many mothers faced challenges accessing the CHC-based universal services.

#### SMS messages and booklets were useful for enhancing knowledge

Most mothers reported usefulness of the program SMS messages and booklets: *“[the booklets] were all useful and they all contained useful information”* (ID22; Arabic; multiparous; 8 years)*.* Staff all endorsed the general usefulness and content of the booklets. One staff member reflected on her own experience: *“Like for me, each time I look at these booklets, I said ‘wow, that’s really perfect’ because I would get pregnant here if this was the kind of service”* – and went on to say that the booklets could be enhanced: *“Sometimes women ask for information which is not in the booklet […] if you include more information it will be more excellent”* (Project assistant; Arabic, English). The suggested additional content included formula feeding, allergies and infant feeding during Ramadan (a month of fasting that is practised in Islam).

Most consistently, mothers highlighted the most useful content related to breastfeeding, baby cues, responsive feeding, tummy-time, sleep and settling. Arabic-speaking mothers more commonly also highlighted the value of learning about communicating with baby, related to responsive practices: *“This is the first time I find this information […] the main thing was the sleeping time for the baby and how to understand signs when the baby needs something”* (ID18; Arabic; multiparous; 11 years). Many mothers, especially first-time mothers, wanted more detailed information about introducing solids, including recipes. Several mothers, with a range of years living in Australia, talked about learning the importance of timing for introducing solids, however, there was still some confusion. Starting solids around 6 months was often different to what they had heard from their doctor or friends and family (which was usually earlier, at 4–5 months) (relates to the previous theme).

Mothers found that generally the SMS messages were a useful reminder and prompt alongside the nurse calls and booklets. *“I like the SMS, I find that it’s helpful, because it sort of serves as a reminder, reminding of what is happening, what to do, because it usually matches the age of development, so I know what to do with the baby’s age*” (ID3; Chinese; first-time; 4 years). However, some mothers, with multiple children particularly, felt that the SMS messages were too simple and brief: *“There wasn’t much information in the SMS, like for example, ‘breast feeding is good for the baby’”* (ID13; Arabic; multiparous; 7 years).

#### Perceptions of the program being especially useful for first-time mothers and new migrants

Some mothers spoke of the Healthy Beginnings program being especially important for first-time mothers who are new to motherhood: *“For me it more served as a reminder, maybe for the first-time mothers it would be more useful”* (ID2; Chinese; multiparous; 5 years). Staff also perceived that the service was particularly relevant for first-time mothers: *“I find that the first-time mothers tend to be more motivated. And they need the service more than others and they would follow advice through”* (CFHN; Arabic, English). Yet, mothers with multiple children also commented on how they found the program useful and many gave examples of increased knowledge and behaviour changes particularly for introducing solids, settling techniques and tummy-time practices: *“So this is the first time I find this information, useful information, in regard to caring for the baby - the main thing was the sleeping time for the baby and how to understand signs when the baby needs something”* (ID18; Arabic; multiparous; 11 years). These mothers were also able to make comparisons with their previous experiences; several mothers talked about noticing increased strength of their baby (participating in the program) as a result of tummy-time practices: *“Tummy-time is really useful for my second baby; he is very strong compared to my daughter”* (ID8; Chinese; multiparous; 11 years).

A few mothers in both language groups, particularly those living in Australia for a longer period, said the program may be more useful to mothers more newly-arrived in Australia who experience language barriers and limited social support: “*The new mum who is new in this country and doesn’t have any relatives or who really, really struggle”* (ID11; Arabic; multiparous; 10 years). Many staff also expressed this opinion: *“Some of them were just married and this is their first child, and they don’t have enough experience. They just came to Australia. When you are on your own without family, without parents, so you start to worry, how will I get this information […] So when they find this information, they felt comfortable”* (Project assistant; Arabic, English).

### Construct 4: Burden

#### Logistical issues limited mothers’ participation

Some mothers who received fewer nurse calls commented on not being able to talk to the nurse for reasons such as nurse availability (1 day per week), call scheduling, and uncertainty of contact numbers. Mothers offered suggestions related to bi-cultural CFHN availability: “*If there are more available times or opportunities that I can talk that will be much better, because I like to talk with [the nurse], and I think she only works on Thursdays”* (ID19; Chinese; first-time; 9 years). Suggestions were also offered related to scheduling: *“[the program] could be improved a little bit better if they tell the times the nurse would be available. I’m not sure what time I need to contact her”* (ID11; Arabic; multiparous; 10 years). One mother was not satisfied with the response time from the nurses: “I *understand that I can organise the nurse to call, but for me, I think that waiting for one to two days is a bit too long”* (ID193; Chinese; multiparous; 5 years). Mothers who received all four nurse calls did not comment on any logistical issues or burdens to participation. Program delivery staff suggested more staff time for the nurse calls: “*I know that the telephone support calls became less frequent, I think that was only because it was an issue of staff. I think that the participants really benefited from the telephone support calls, they really enjoyed them, I think it was the standout”* (Project assistant; Arabic, English).

#### The phone-based service was seen as convenient

Most mothers described the phone-based support as convenient for them. They mentioned there being no need to pre-book, wait in a waiting room nor arrange transport. Some mothers made comparisons with visiting a clinic: *“I have two babies and I’ve not had the time to go the doctor or a nurse to ask about questions but through the booklets I’ve learned about the development stages of the baby because I used to receive them every month, like as the baby was growing.”* (ID4; Arabic; multiparous; 1.5 years). One mother commented that the phone-based service made her more comfortable: *“Because I can ask any questions, and I can book a time with her more suited to my time. I don’t have to go to a particular clinic […] I can still ask questions; I am more relaxed and more comfortable”* (ID8; Chinese; multiparous; 11 years). Program delivery staff also spoke of the convenience of the nurse calls and having more time to talk with mothers: *“Usually in a Centre you’d be assessing the mother and the baby, like you’d be talking to the mum, but you’d be weighing and measuring the baby and stuff like that as well. Whereas on the phone you can concentrate on what you’re doing”* (CFHN; English).

#### Mothers are busy and do not always have time

Some mothers did not find it easy to participate in the program because they felt time-poor and busy with their new baby irrespective of how many children they had: “*After birth, for 1-2 months, I was very busy, I did not have time to follow [the advice]. I haven’t got time; everything was a mess”* (ID12; Chinese; first-time; 1.5 years). Mothers made comments about time for support generally too: *“I know there is a local clinic that I can go to, but because of how busy we are as mums …we don’t have time to take the baby and go to the clinic whenever we have a question”* (ID3; Arabic; multiparous; 17 years). Staff also noted this: *“mothers when they have new babies, they don’t have time to talk […] the majority, they put their family life first.”* (Project assistant; Arabic, English). The support calls are primarily for establishing health behaviours as opposed to resolving urgent problems, and one of the nurses reflected on this: *“I feel that adult learning is more sort of purposeful orientated, so we don’t just learn anything we learn something that we want to learn. So, as a parent, when they have a baby they say, ‘oh, my gosh, I don’t know, what should I do’, that’s actually the time that they search for the information*” (CFHN; Mandarin, English). This relates not only to time for the program involvement, but motivations and relevance of the program to mothers’ needs at that time.

### Construct 5: Intervention coherence

#### Uncertainties about the program impacted involvement

There were times where mothers, particularly those who completed fewer nurse calls, were not sure about how the program worked and so engaged less in the program. For example, some mothers were not sure whether the program offered a helpline for immediate support. One Arabic-speaking mum who did not complete any nurse calls was expecting more support: “*When I signed up, I expected more involvement and face-to-face involvement”* (ID3; Arabic; multiparous; 17 years). Other comments included uncertainty about whether the SMS messages were two-way and whether the messages were automated: *“I thought the SMS message was just artificial, no human behind an automated message”* (ID13; Chinese; first-time; 6.5 years). A staff member reported this also: *“Sometimes I talk to the mothers, and then they didn’t know that they can actually send SMS to ask to speak to the nurse”* (Project officer; Mandarin, Cantonese, English). One mother, who did not receive calls, did not engage with the program fully she was not sure about the nurse’s language: *“I wasn’t sure whether the nurse can speak Chinese”* (ID18; Chinese, multiparous; 5 years). The staff also found that some mothers were not sure of how the program worked and suggested more information be given at recruitment and throughout the program.

## Discussion

### Key findings

Our mixed methods findings demonstrate feasibility of the culturally adapted Healthy Beginnings program delivered by telephone, promoting early life nutrition and active play behaviours, and strong acceptability among participating Arabic- and Chinese-speaking mothers. Our study outcomes were favourable, affirming the preceding systematic cultural adaptation stages we undertook to modify the program for cultural relevance [29]. This study provides valuable learning for refining the culturally adapted program and preparing for a future trial. This study also offers insights for early life behavioural interventions among culturally and linguistically diverse migrant mothers.

Our retention rate (83%) is comparable to the mainstream Healthy Beginnings CHAT trial at 6-months among mothers who spoke a language other than English at home (79%) [26]. Our retention rate is favourable when compared to other international culturally adapted early obesity prevention interventions [48, 49]. Mothers completed more of the earlier nurse calls (antenatal and 1 month) compared to the later ones (3 and 5 months), which also aligns with findings in the mainstream Healthy Beginnings CHAT trial [26]. Compared with our results, other culturally adapted early life obesity prevention programs show similar [49] and less favourable intervention engagement [48], however comparisons are challenging as there are few similar programs.

### Factors that enhanced feasibility outcomes

From our mixed methods findings, several key factors contributed to the feasibility of the culturally adapted Healthy Beginnings program among participating Arabic- and Chinese-speaking mothers.

#### Uniqueness of the program – culturally relevant, specialised support

Generally, mothers valued the program, particularly that it was linguistically matched with culturally adapted written resources and with personalised support from bi-cultural CFHNs. Our formative research supported the importance of culturally sensitive care for mothers [30]. Other early childhood obesity prevention interventions highlight the importance of culturally relevant care [50, 51] and parents’ expressed need for specialised infant feeding and active play advice [52]. Universal child and family health services in Australia do include nutrition and active play, however there are many important topics to cover (e.g., physical assessments, safety), and CFHNs report time constraints and staff shortages as barriers to delivering comprehensive services [35]. Consistent with our findings, mothers have reported doctors consultations to sometimes be rushed and lacking specialist infant advice [53]. Differences in language and cultural background of health professionals and recipients is also an important consideration for engagement [8, 54]. Many mothers in this study felt they were given time for the interaction and were emotionally supported by the bi-cultural CFHNs.

#### Accessibility of the telephone support

For many participating mothers, the regular telephone support from bicultural CFHNs was valued and convenient. In the mainstream Healthy Beginnings program (CHAT), the nurse calls were also appreciated [55] and associated with the strongest behavioural outcomes [25]. Almost all mothers in this study, and in our formative study [30], irregularly accessed universal child and family health services. Mothers from diverse cultural backgrounds can experience barriers to accessing universal child and family health services, such as transportation, language, costs and uncertainty with making appointments [56]. The culturally adapted Healthy Beginnings program overcame many of these issues. Continuity of care and outreach services are known avenues for supporting migrant mothers to access child and family health services [56, 57]. Health provider interactions are important for trust and ongoing engagement with culturally diverse clients [58].

#### Participant sociodemographic characteristics

There were demographic differences among mothers who participated in this study, which may partially explain some variation in feasibility outcomes. This also relates to intersectionality theory and the need to consider multiple dimensions of identity in health promotion interventions [59]. Our qualitative acceptability findings highlighted that mothers and staff perceived the program to be more useful for first-time mothers compared to mothers with multiple children, and the quantitative satisfaction data supported this. The need for information as a first-time parent has been identified as an enabler to engagement in a similar early obesity prevention program [52]. Other studies indicate that birth order (the order in which a child is born in a family) may influence breastfeeding practices, investment in infant health [60] and early parenting intervention effectiveness [61].

Our findings also indicate greater engagement and retention among mothers who had more recently arrived in Australia. Newer migrants are in the early stages of adapting to a new country and culture [62], therefore may be in greater need of culturally appropriate support [5, 63]. This aligns with our formative qualitative research findings [30] and other meta-syntheses [14, 15] describing the challenge of merging traditional feeding practices with the dominant culture after migration. Newer migrants’ access to health services may be impacted by unfamiliarity with the healthcare system and other factors such as income, family situation, reasons for migration, pre-migration experiences and social capital [64].

#### Implementation factors

The follow-up interviews revealed that mothers who expressed uncertainties about the program were generally less satisfied with their experiences. Staff interviews endorsed the need to enhance the description of program involvement at recruitment. This aligns with recruitment experiences for other culturally adapted early life interventions [49]. Providing a concise written summary of the commitment and offering information sessions through established groups and trusted community organisations have been successful in previous studies [65, 66]. The constraints of project budget and staffing also impacted program delivery staff (bi-cultural CFHNs) availability, which in turn impacted engagement in nurse calls and also acceptability of the program.

### The cultural adaptation process and next stages

The Stages of Cultural Adaptation (Fig. 1) was used to guide the process of culturally adapting the Healthy Beginnings program delivered by telephone. Using an established cultural adaptation model resulted in a highly relevant and acceptable program, with detailed reporting to assist others with this methodology. The promising outcomes of this feasibility study (representing Stage 4, Fig. 1) indicate that the cultural adaptations undertaken in Stages 1–3 [29] enhanced the program’s cultural relevance among the new target populations: Arabic- and Chinese-speaking migrant mothers. These feasibility findings inform Stages 5 and 6: refinements to enhance program adaptations, and a trial to investigate effectiveness.

Table 4 outlines opportunities to refine the program, including increasing bi-cultural CFHN availability, clarifying participant involvement at the time of recruitment, and specific content adjustments. Factors that require further consideration and resources include how to engage participants’ family members and development of a culturally relevant and in language website or app to present program content.

**Table 4:** Proposed refinements to the culturally adapted Healthy Beginnings program

Key area	Proposed refinements
Set-up and preparation	• Ensure adequate time and resources for set-up (e.g. administrative systems) • Finalise operational procedures and provide training to all staff • Ensure bicultural and bilingual staff from target communities are part of the project team
Recruitment	▪ Enhance strategies to describe the study at time of recruitment (e.g. a short video, a handout summarising the plain language statement, a media story to promote benefits of participating) ▪ Convey the key purpose of the program to potential participants (e.g. health department offering free parenting support by a nurse in your language)
Program delivery	• Ensure adequate bi-cultural nursing staff availability • Plan for longer nurse call consultation time, particularly for mothers with complex situations • Consider ways to increase flexibility of call scripts and call script data entry • Create a resource list for nurses to use during calls, including videos/materials for visuals as needed • Consider ways to increase involvement of participant’s family in the program • Continue to send program resources to participants by mail, but also send by email. Consider other online formats for program delivery. • Actively link with and refer to community services – e.g., general practitioners, health care services, playgroups and cultural community organisations
Program content	• Strengthen messaging regarding introducing solids at around 6 months of age in the program booklets • Include additional information about baby development, food textures and recipes, baby sleep, tired signs, and best-practice formula feeding • Include information about mothers’ diet and infant feeding during cultural events • Enhance sections about mothers’ mental health, support, and self-care practices

Based on our results, a trial to assess the effectiveness and cost-effectiveness of the culturally adapted Healthy Beginnings program is warranted (relates to Stage 6, Fig. 1). The design of this proposed trial would ideally allow for direct comparison between a control group, the mainstream and the culturally adapted Healthy Beginnings to assess the program’s impact on key modifiable feeding and play behaviours and child weight. A larger sample size would also allow us to investigate associations with a range of individual characteristics. Establishing a-priori progression criteria for this proposed trial will be important to determine implementing a larger trial or scale-up [67].

### Implications for policy and practice

The culturally adapted Healthy Beginnings program has potential to be embedded into universal child and family health services for supporting the wellbeing of mothers and their infants. The program uses strategies for engaging culturally and linguistically diverse families that align with those suggested in Australia’s national framework for universal child and family health services [68] (such as tailored services, proactive outreach and culturally-specific programs), however in practice these are inconsistently operationalised. Sustained investment in appropriate resources (including staff, time and funding) and building the capacity of services and staff to support culturally and linguistically diverse families are required to action such strategies and improve health outcomes among migrant families. From our findings, we also suggest pragmatic strategies such as enhancing continuity of care and connecting with bi-cultural doctors and local migrant organisations to increase culturally and linguistically diverse migrant mothers’ access to child and family health services.

### Study strengths and limitations

To our knowledge, this is the first Australian cultural adaptation of an intervention for early childhood obesity prevention. Engaging with bi-cultural and bi-lingual workforce is a critical strength of this study and the project. The individualised support for mothers from bi-cultural and bilingual expert nurses offers potential for improved health service uptake as well as improved target behaviours. We recruited a large sample for this feasibility study and had a high retention rate, indicating positive uptake from participants and greater representation from the target groups. Our qualitative interviews and acceptability analysis were a strength of this study, particularly as acceptability is a significant enabler for child and family health service access among linguistically diverse migrants [8] and participants’ experiences in early childhood obesity prevention studies are infrequently reported [69]. The qualitative follow-up interviews conducted allowed us to explore mothers’ program experiences in detail. The Theoretical Framework of Acceptability [42] was a useful tool to guide a structured investigation and identify specific issues within acceptability constructs.

This was a feasibility study with resource constraints and therefore we were limited with how much implementation data we could collect. It would have been valuable to also capture the duration of nurse calls, nurse adherence to the call scripts and the number of ad-hoc participant interactions with nurses. While efforts were made to reach a range of mothers for the follow-up interviews, we mostly interviewed mothers who completed more nurse calls and who may therefore have had more positive experiences with the program. Follow-up interviews with mothers were conducted in two languages (with a project team member in English and a co-facilitator interpreter) with only the English dialogue transcribed due to funding constraints. This may have limited the depth of the interview and analysis. To minimise participant burden, surveys were short, and we did not ask mothers about additional characteristics (such as their self-rated English language proficiency or whether this was their first birth in Australia), which could have provided further insights about program engagement.

## Conclusion

The culturally adapted Healthy Beginnings program [29] for promoting early life nutrition and active play behaviours was feasible to deliver and acceptable among participating Arabic- and Chinese-speaking migrant mothers in Australia. Our results highlight the importance of culturally adapted and in-language resources, individualised bi-cultural nurse support and telephone-based service delivery for supporting culturally and linguistically diverse migrant families with optimal infant feeding and active play during the pre- and post-natal period. Study findings will inform future refinements to enhance reach and delivery of the culturally adapted program, and also offer a strong foundation for a future trial to assess program effectiveness and scalability.

## Supplementary Information

**Additional file 1.** CONSORT reporting checklist. CONSORT 2010 checklist of information to include when reporting a pilot or feasibility trial.

**Additional file 2.** Program participant surveys administered by phone. These are the English versions of the surveys with mothers before the program (28–36 weeks gestation) and after the program (infant age 6-months). The surveys were professionally translated and reviewed, then trained bi-cultural research staff administered the surveys in language over the phone with mothers.

**Additional file 3.** Interview guide for follow-up with participating mothers. English version of the interview guide with mothers who had participated in the program. Interviews were conducted in language and English. Interviews were co-facilitated by English-speaking staff members and with a bi-lingual staff member or one of two professional interpreters.

**Additional file 4.** Brief survey and interview guide for follow-up with staff. Brief survey and interview guide with staff who implemented the program. Interviews were conducted in English.

**Additional file 5.** Mothers’ satisfaction scores. Participant satisfaction with overall program experience using the Client Satisfaction Questionnaire (CSQ-8) measured at the 6-month survey. Bivariate analyses to assess association between potential explanatory factors and the satisfaction score.

**Additional file 6.** Demographic characteristics of mothers involved in interviews. Demographic characteristics of mothers who agreed to be contacted for an interview at program completion, and those who competed a follow-up interview.

## Data Availability

The de-identified data that support the findings of this study may be available upon reasonable request to the corresponding author SM and with ethics approval.

## Abbreviations

AUD: Australian dollars
CFHN: Child and family health nurse
CHAT: Communicating Healthy Beginnings Advice by Telephone
ID: Identification
RCT: Randomised controlled trial
SMS: Short message service
TFA: Theoretical Framework of Acceptability

## References

[1] De Freitas C, Massag J, Amorim M, Fraga S (2020). Involvement in maternal care by migrants and ethnic minorities: a narrative review. Public Health Rev.

[2] Au M, Anandakumar AD, Preston R, Ray RA, Davis M (2019). A model explaining refugee experiences of the Australian healthcare system: a systematic review of refugee perceptions. BMC Int Health Hum Rights.

[3] Benza S, Liamputtong P (2014). Pregnancy, childbirth and motherhood: a meta-synthesis of the lived experiences of immigrant women. Midwifery..

[4] United Nations (2017). International migration report 2017.

[5] Ou L, Chen J, Hillman K (2010). Health services utilisation disparities between English speaking and non-English speaking background Australian infants. BMC Public Health.

[6] Yelland J, Riggs E, Small R, Brown S (2015). Maternity services are not meeting the needs of immigrant women of non-English speaking background: results of two consecutive Australian population based studies. Midwifery..

[7] Ogbo FA, Eastwood J, Hendry A, Jalaludin B, Agho KE, Barnett B, et al. Determinants of antenatal depression and postnatal depression in Australia. BMC Psychiatry. 2018;18(1):49 10.1186/s12888-018-1598-x. 10.1186/s12888-018-1598-x PMC5819705 29463221

[8] Dougherty L, Lloyd J, Harris E, Caffrey P, Harris M (2020). Access to appropriate health care for non-English speaking migrant families with a newborn/young child: a systematic scoping literature review. BMC Health Serv Res.

[9] Victora CG, Bahl R, Barros AJ, França GV, Horton S, Krasevec J, et al. Breastfeeding in the 21st century: epidemiology, mechanisms, and lifelong effect. Lancet. 2016;387(10017):475–90 10.1016/S0140-6736(15)01024-7. 10.1016/S0140-6736(15)01024-7 26869575

[10] Haszard JJ, Russell CG, Byrne RA, Taylor RW, Campbell KJ (2019). Early maternal feeding practices: associations with overweight later in childhood. Appetite..

[11] Woo Baidal JA, Locks LM, Cheng ER, Blake-Lamb TL, Perkins ME, Taveras EM (2016). Risk factors for childhood obesity in the first 1,000 days: a systematic review. Am J Prev Med.

[12] Hewitt L, Kerr E, Stanley RM, Okely AD (2020). Tummy time and infant health outcomes: a systematic review. Pediatrics..

[13] Dennis CL, Shiri R, Brown HK, Santos HP, Schmied V, Falah-Hassani K (2019). Breastfeeding rates in immigrant and non-immigrant women: a systematic review and meta-analysis. Matern Child Nutr.

[14] Joseph J, Brodribb W, Liamputtong P (2019). “Fitting-in Australia” as nurturers: meta-synthesis on infant feeding experiences among immigrant women. Women Birth.

[15] Schmied V, Olley H, Burns E, Duff M, Dennis CL, Dahlen HG (2012). Contradictions and conflict: a meta-ethnographic study of migrant women’s experiences of breastfeeding in a new country. BMC Pregnancy Childbirth.

[16] Hardy LL, Jin K, Mihrshahi S, Ding D (2019). Trends in overweight, obesity, and waist-to-height ratio among Australian children from linguistically diverse backgrounds, 1997 to 2015. Int J Obes.

[17] Blake-Lamb TL, Locks LM, Perkins ME, Woo Baidal JA, Cheng ER, Taveras EM (2016). Interventions for childhood obesity in the first 1,000 days: a systematic review. Am J Prev Med.

[18] Wen LM, Baur LA, Rissel C, Wardle K, Alperstein G, Simpson JM (2007). Early intervention of multiple home visits to prevent childhood obesity in a disadvantaged population: a home-based randomised controlled trial (healthy beginnings trial). BMC Public Health.

[19] Campbell K, Hesketh K, Crawford D, Salmon J, Ball K, McCallum Z (2008). The Infant Feeding Activity and Nutrition Trial (INFANT) an early intervention to prevent childhood obesity: cluster-randomised controlled trial. BMC Public Health.

[20] Daniels LA, Magarey A, Battistutta D, Nicholson JM, Farrell A, Davidson G, et al. The NOURISH randomised control trial: positive feeding practices and food preferences in early childhood - a primary prevention program for childhood obesity. BMC Public Health. 2009;9(1):387. 10.1186/1471-2458-9-387. 10.1186/1471-2458-9-387 PMC2770488 19825193

[21] Askie LM, Espinoza D, Martin A, Daniels LA, Mihrshahi S, Taylor R, et al. Interventions commenced by early infancy to prevent childhood obesity—The EPOCH Collaboration: An individual participant data prospective meta-analysis of four randomized controlled trials. Pediatr Obes. 2020:e12618 10.1111/ijpo.12618. 10.1111/ijpo.12618 32026653

[22] Wen LM, Baur LA, Simpson JM, Rissel C, Wardle K, Flood VM (2012). Effectiveness of home based early intervention on children's BMI at age 2: randomised controlled trial. BMJ..

[23] Wen LM, Baur LA, Simpson JM, Rissel C, Flood VM (2011). Effectiveness of an early intervention on infant feeding practices and “tummy time”: a randomized controlled trial. Arch Pediatr Adolesc Med.

[24] Wen LM, Rissel C, Baur LA, Hayes AJ, Xu H, Whelan A, et al. A 3-arm randomised controlled trial of Communicating Healthy Beginnings Advice by Telephone (CHAT) to mothers with infants to prevent childhood obesity. BMC Public Health. 2017;17(1):79 10.1186/s12889-016-4005-x. 10.1186/s12889-016-4005-x PMC5237545 28088203

[25] Wen LM, Rissel C, Xu H, Taki S, Buchanan L, Bedford K, et al. Effects of telephone and short message service support on infant feeding practices, “tummy time,” and screen time at 6 and 12 months of child age: A 3-group randomized clinical trial. JAMA Pediatr. 2020:e200215 10.1001/jamapediatrics.2020.0215. 10.1001/jamapediatrics.2020.0215 PMC7154951 32282034

[26] Marshall S, Taki S, Love P, Laird Y, Kearney M, Tam N, et al. Engagement, satisfaction, retention and behavioural outcomes of linguistically diverse mothers and infants participating in an Australian early obesity prevention trial. Heal Promot J Aust. 2021; Under review. 10.1002/hpja.521 34245623

[27] Australian Bureau of Statistics. 2016 Census. 2020. [cited 15 March 2021]. Available from: https://quickstats.censusdata.abs.gov.au/census_services/getproduct/census/2016/quickstat/1GSYD.

[28] Barrera M, Castro FG, Strycker LA, Toobert DJ (2012). Cultural adaptations of behavioral health interventions: a progress report. J Consult Clin Psychol.

[29] Marshall S, Taki S, Love P, Laird Y, Kearney M, Tam N, et al. The process of culturally adapting the healthy beginnings early obesity prevention program for Arabic and Chinese mothers in Australia. BMC Public Health. 2021;21(1):284 10.1186/s12889-021-10270-5. 10.1186/s12889-021-10270-5 PMC7863271 33541310

[30] Marshall S, Taki S, Love P, Kearney M, Tam N, Sabry M, et al. Navigating infant feeding supports after migration: Perspectives of Arabic and Chinese mothers and health professionals in Australia. Women Birth. 2020:S1871–5192(20)30267–5 10.1016/j.wombi.2020.06.002. 10.1016/j.wombi.2020.06.002 32600988

[31] Moore GF, Audrey S, Barker M, Bond L, Bonell C, Hardeman W, et al. Process evaluation of complex interventions: Medical Research Council guidance. BMJ. 2015;350:h1258 10.1136/bmj.h1258. 10.1136/bmj.h1258 PMC4366184 25791983

[32] Movsisyan A, Arnold L, Evans R, Hallingberg B, Moore G, O’Cathain A, et al. Adapting evidence-informed complex population health interventions for new contexts: a systematic review of guidance. Implement Sci. 2019;14(1):105 10.1186/s13012-019-0956-5. 10.1186/s13012-019-0956-5 PMC6918624 31847920

[33] Eldridge SM, Chan CL, Campbell MJ, Bond CM, Hopewell S, Thabane L, et al. CONSORT 2010 statement: extension to randomised pilot and feasibility trials. BMJ. 2016;355:i5239 10.1136/bmj.i5239. 10.1136/bmj.i5239 PMC5076380 27777223

[34] Lancaster GA, Thabane L (2019). Guidelines for reporting non-randomised pilot and feasibility studies. Pilot Feasibility Stud.

[35] Schmied V, Fowler C, Rossiter C, Homer C, Kruske S, CHoRUS team (2014). Nature and frequency of services provided by child and family health nurses in Australia: results of a national survey. Aust Health Rev.

[36] Healthy Beginnings. 2019. [cited 15 March 2021]. Available from: https://www.healthybeginnings.net.au/

[37] Billingham SA, Whitehead AL, Julious SA (2013). An audit of sample sizes for pilot and feasibility trials being undertaken in the United Kingdom registered in the United Kingdom clinical research network database. BMC Med Res Methodol.

[38] Harris PA, Taylor R, Thielke R, Payne J, Gonzalez N, Conde JG (2009). Research electronic data capture (REDCap)--a metadata-driven methodology and workflow process for providing translational research informatics support. J Biomed Inform.

[39] Beaton DE, Bombardier C, Guillemin F, Ferraz MB (2000). Guidelines for the process of cross-cultural adaptation of self-report measures. Spine (Phila Pa 1976).

[40] Suurmond J, Woudstra A, Essink-Bot ML (2016). The interpreter as co-interviewer: the role of the interpreter during interviews in cross-language health research. J Health Serv Res Policy.

[41] Bowen DJ, Kreuter M, Spring B, Cofta-Woerpel L, Linnan L, Weiner D, et al. How we design feasibility studies. Am J Prev Med. 2009;36(5):452–7 10.1016/j.amepre.2009.02.002. 10.1016/j.amepre.2009.02.002 PMC2859314 19362699

[42] Sekhon M, Cartwright M, Francis JJ (2017). Acceptability of healthcare interventions: an overview of reviews and development of a theoretical framework. BMC Health Serv Res.

[43] Attkisson CC, Zwick R (1982). The client satisfaction questionnaire. Psychometric properties and correlations with service utilization and psychotherapy outcome. Eval Program Plann.

[44] Morgan D. Pragmatism as a paradigm for mixed methods research. In: Integrating qualitative and quantitative methods: SAGE Publications, Inc; 2014. p. 25–44. 10.4135/9781544304533.

[45] IBM Corp (2017). IBM SPSS Statistics for Windows, Version 25.

[46] Gale NK, Heath G, Cameron E, Rashid S, Redwood S (2013). Using the framework method for the analysis of qualitative data in multi-disciplinary health research. BMC Med Res Methodol.

[47] Morgan D (2007). Paradigms lost and pragmatism regained: methodological implications of combining qualitative and quantitative methods. J Mixed Methods Res.

[48] Pallan M, Hurley KL, Griffin T, Lancashire E, Blissett J, Frew E, et al. A cluster-randomised feasibility trial of a children's weight management programme: the Child weigHt mANaGement for Ethnically diverse communities (CHANGE) study. Pilot Feasibility Stud. 2018;4(1):175. 10.1186/s40814-018-0373-6. 10.1186/s40814-018-0373-6 PMC6260774 30505457

[49] McEachan RRC, Santorelli G, Bryant M, Sahota P, Farrar D, Small N (2016). The HAPPY (Healthy and Active Parenting Programmme for early Years) feasibility randomised control trial: acceptability and feasibility of an intervention to reduce infant obesity. BMC Public Health.

[50] Reifsnider E, McCormick DP, Cullen KW, Szalacha L, Moramarco MW, Diaz A, et al. A randomized controlled trial to prevent childhood obesity through early childhood feeding and parenting guidance: rationale and design of study. BMC Public Health. 2013;13(1):880. 10.1186/1471-2458-13-880. 10.1186/1471-2458-13-880 PMC3852597 24063435

[51] Reifsnider E, McCormick DP, Cullen KW, Todd M, Moramarco MW, Gallagher MR, et al. Randomized controlled trial to prevent infant overweight in a high-risk population. Acad Pediatr. 2018;18(3):324–33 10.1016/j.acap.2017.12.007. 10.1016/j.acap.2017.12.007 PMC5889724 29277462

[52] Love P, Laws R, Litterbach E, Campbell KJ (2018). Factors influencing parental engagement in an early childhood obesity prevention program implemented at scale: the infant program. Nutrients..

[53] Corr L, Rowe H, Fisher J (2015). Mothers’ perceptions of primary health-care providers: thematic analysis of responses to open-ended survey questions. Aust J Prim Health.

[54] Levesque JF, Harris MF, Russell G (2013). Patient-centred access to health care: conceptualising access at the interface of health systems and populations. Int J Equity Health.

[55] Ekambareshwar M, Taki S, Mihrshahi S, Baur LA, Rissel C, Wen LM (2020). Participant experiences of an infant obesity prevention program delivered via telephone calls or text messages. Healthcare..

[56] Riggs E, Davis E, Gibbs L, Block K, Szwarc J, Casey S, et al. Accessing maternal and child health services in Melbourne, Australia: reflections from refugee families and service providers. BMC Health Serv Res. 2012;12(1):117. 10.1186/1472-6963-12-117. 10.1186/1472-6963-12-117 PMC3424108 22587587

[57] Handtke O, Schilgen B, Mösko M (2019). Culturally competent healthcare - a scoping review of strategies implemented in healthcare organizations and a model of culturally competent healthcare provision. PLoS One.

[58] Saha S, Beach MC, Cooper LA (2008). Patient centeredness, cultural competence and healthcare quality. J Natl Med Assoc.

[59] Heard E, Fitzgerald L, Wigginton B, Mutch A (2020). Applying intersectionality theory in health promotion research and practice. Health Promot Int.

[60] Buckles K, Kolka S (2014). Prenatal investments, breastfeeding, and birth order. Soc Sci Med.

[61] Stolk MN, Mesman J, van Zeijl J, Alink L, Bakermans-Kranenburg M, van IJzendoorn M, et al. Early parenting intervention: family risk and first-time parenting related to intervention effectiveness. J Child Fam Stud. 2008;17(1):55. 10.1007/s10826-007-9136-3–83.

[62] Berry JW. Theories and models of acculturation. In: Schwartz SJ, Unger J, editors. The Oxford Handbook of Acculturation and Health: Oxford University Press; 2017. 10.1093/oxfordhb/9780190215217.013.2.

[63] Volandes AE, Paasche-Orlow MK (2007). Health literacy, health inequality and a just healthcare system. Am J Bioeth.

[64] Grant J, Guerin PB (2014). Applying ecological modeling to parenting for Australian refugee families. J Transcult Nurs.

[65] Hughson JA, Woodward-Kron R, Parker A, Hajek J, Bresin A, Knoch U, et al. A review of approaches to improve participation of culturally and linguistically diverse populations in clinical trials. Trials. 2016;17(1):263 10.1186/s13063-016-1384-3. 10.1186/s13063-016-1384-3 PMC4880985 27229153

[66] Bhutta ZA (2004). Beyond informed consent. Bull World Health Organ.

[67] Mbuagbaw L, Kosa SD, Lawson DO, Stalteri R, Olaiya OR, Alotaibi A, et al. The reporting of progression criteria in protocols of pilot trials designed to assess the feasibility of main trials is insufficient: a meta-epidemiological study. Pilot Feasibility Stud. 2019;5(1):120. 10.1186/s40814-019-0500-z. 10.1186/s40814-019-0500-z PMC6827233 31700654

[68] Australian Government Department of Health (2011). National Framework for Universal Child and Family Health Services.

[69] Ekambareshwar M, Ekambareshwar S, Mihrshahi S, Wen LM, Baur LA, Laws R, et al. Process evaluations of early childhood obesity prevention interventions delivered via telephone or text messages: a systematic review. Int J Behav Nutr Phys Act. 2021;18(1):10 10.1186/s12966-020-01074-8. 10.1186/s12966-020-01074-8 PMC7796572 33422066

