# Supplementary material for: Feasibility of a culturally adapted early childhood obesity prevention program among migrant mothers in Australia: a mixed methods evaluation
Source: BMC Public Health. 2021 Jun 16;21:1159. doi: 10.1186/s12889-021-11226-5 (PMC8207722; doi:10.1186/s12889-021-11226-5)
Supplement: Supplementary file 2 — Additional file 2. Program participant surveys administered by phone. These are the English versions of the surveys with mothers before the program (28–36 weeks gestation) and after the program (infant age 6-months). The surveys were professionally translated and reviewed, then trained bi-cultural research staff administered the surveys in language over the phone with mothers. [file 12889_2021_11226_MOESM2_ESM.docx]

## Additional file 2: Program participant surveys administered by phone

These are the English versions of the telephone surveys with mothers. The surveys were professionally translated and reviewed, then trained bi-cultural research staff administered the surveys in in Chinese or Arabic languages over the phone with mothers.

- Survey 1: At baseline/before the program (at 28-36 weeks gestation) – pages 1-12
- Survey 2: At the end of the program (at infant age 6-months) – pages 13-30

**Communicating Healthy Beginnings Advice by Telephone (CHAT): for Arabic or Chinese speaking Mothers**

**Mothers/ Pregnant Women Baseline Telephone Survey**

***Please read this section to participant*:**

Hi__________________________ (participant name)

My name is ______, calling on behalf of Health Beginnings which you recently registered to participate in, from [location] on [date/day].

You would have been told that someone from the project would be calling to collect some initial information about you to help with the study. The survey will take approximately 15-30 minutes to complete.

When we complete the survey we will arrange to send you your choice of a Coles or Woolworths giftcard to the value of $20.

This call may be recorded for quality control and training purposes. The answers that you give are confidential and are only used for statistical purposes in combination with all the other participants in the survey. Your identity will not be disclosed to any other organisations.

If you’d like to know more about how we protect your privacy, I will organise for our Project Coordinator from the Health Promotion Unit Sydney Local Health District to contact you or can provide you with her telephone details (02 9515 9075).

First, I will ask you questions about your pregnancy, then about your physical activity and nutrition, and about your baby.

**[Survey introduction]**

S1 Is now a convenient time for us to go through them, it would be helpful if you could be in a quiet place to talk?

**[DO NOT READ RESPONSE OPTIONS]**

1. Yes 🡪 SKIP TO S4
2. Yes, but not now 🡪 ARRANGE CALLBACK

When is a good day/time for me to ring back?

___________day _________am / pm

1. No, I would like to withdraw 🡪 CONTINUE TO S2

S2 No problem, is there any particular reason why you would like to withdraw from the study?

**[DO NOT READ RESPONSE OPTIONS]**

1. Miscarriage
2. Stillborn
3. Other Please specify __________________________________

S3 We will let the Healthy Beginnings team know. We are sorry for your loss. Do you need any assistance from the hospital or other service?

|  |
| --- |
|  |
|  |

[**END SURVEY**] ADVISE SUPERVISOR IMMEDIATELY.

S4 Before we get started, if you don’t feel comfortable answering any question we can move on to the next one, this will not affect your eligibility for your gift voucher. Do you have any questions so far? If any questions arise during this interview, we’ll have time to discuss them at the end.

[RECORD RESPONDENT QUESTIONS HERE – DO NOT ANSWER ANY QUESTIONS OTHER THAN THOSE RELATING TO THIS SURVEY THAT YOU ARE APPROVED TO ANSWER.]

[FOR ANY HEALTH-RELATED QUESTIONS SAY] I have made a note of that and the Healthy Beginnings team will contact you to assist.

|  |
| --- |
|  |
|  |

**[Pregnancy status – screening]**

I’d like to start by asking a few questions about your pregnancy. When you registered to Healthy Beginnings program you mentioned that you were PIPE FROM SAMPLE weeks pregnant and your estimated baby’s due date was on PIPE FROM SAMPLE.

1. a) Since registering, can you confirm what your current status is:

- Currently pregnant🡪 Go to Q2
- Gave birth🡪 Go to Q1b
- Other, please specify__________________ **[DO NOT READ i.e. miscarriage, still birth.** **IF still birth or miscarriage need to END SURVEY] 🡪 return to S3**

1 b) Congratulations, could you please give me the birth date of your baby?

__ __ [date] __ __ [month] __ __ __ __ [year]

1 c) Did you have a boy or a girl?

1. Boy
2. Girl

1 d) Can you please tell me your baby’s name? __________

1. How many weeks pregnant are you? _____________weeks
2. How many babies have you had before this pregnancy?_________**[DO NOT READ OUT: Exclude stillbirths]**

**[Mother’s Weight and height]**

1. Approximately how much did you weigh before becoming pregnant?

________ kg OR ________ lbs

- - Don’t know [DO NOT READ]
  - Refused [DO NOT READ]

1. Currently, how much do you weigh?

________ kg OR ________ lbs

- - Don’t know [DO NOT READ]
  - Refused [DO NOT READ]

1. How tall are you without shoes? [Give answer to the nearest cm or inch]

________ cm OR ________ feet ________ inches

- - Don’t know [DO NOT READ]
  - Refused [DO NOT READ]

1. Before you became pregnant, did you consider yourself to be:

1 Acceptable weight

2 Underweight

3 Overweight

4 Don’t know [*DO NOT READ*]

5 Refused [*DO NOT READ*]

IF Q5 AND/OR Q6 = DON’T KNOW, ASK:

Q5a/Q6a As you were unsure or did not know your height or weight would you be able to measure yourself now or provide us with that information when we ring you back in the next few days?

1 Yes, measure now SKIP BACK TO Q5

2 Yes, call me back later CONTINUE (WHEN CALLBACK COMPLETED DATA WILL BE EDITED BACK IN)

3 Refused [*DO NOT READ*]

**[Mother’s sedentary behaviours]**

The following two questions are about your average weekly amount of time using screens and sitting. [INTERVIEWER NOTE: BE SPECIFIC WITH ASKING ABOUT HOURS OR MINUTES]

1. **Currently**, on average, how many hours per day or per week do you spend sitting watching television, videos, DVDs, playing computer or video games, or surfing the internet for pleasure?

________ Hours per day OR ________ Hours per week

- Rarely or never
- Don’t know [*DO NOT READ*]
- Refused [*DO NOT READ*]

1. On average, during the last 7 days, how much time did you usually spend sitting on a weekday? [NOTE: On ONE typical week day]

________ Hours ________Minutes

- Don’t know [*DO NOT READ*]
- Refused [*DO NOT READ*]

**[Mother’s Dietary Behaviours]**

The next few questions are about food. I’m going to read you a list of different foods and drinks. Please tell me how much of each you usually consume per day or per week. [INTERVIEWER NOTE: ENSURE YOU SPECIFY WHAT A SERVE EQUALS IN THE NEXT TWO QUESTIONS.]

1. How many serves of **vegetables** do you usually eat each day? (Include fresh, dried, frozen and tinned vegetables. One serve = ½ cup cooked or 1 cup of salad vegetables)

______serves per day

______serves per week

- Don’t eat vegetables
- Don’t know [*DO NOT READ*]
- Refused [*DO NOT READ*]

1. How many serves of **fruit** do you usually eat each day? (Include fresh, dried, frozen and tinned fruit. One serve = 1 medium piece or 2 small pieces of fruit or 1 cup of diced pieces)

______serves per day

______serves per week

- Don’t eat fruit
- Don’t know [*DO NOT READ*]
- Refused [*DO NOT READ*]

1. How often do you have meals or snacks such as burgers, pizza, chicken, or chips from places like McDonalds, Hungry Jacks, Pizza Hut, KFC, Red Rooster or other takeaway food places?

______times per week

______times per month

- Rarely or never
- Don’t know [*DO NOT READ*]
- Refused [*DO NOT READ*]

The following question does not include diet drinks.

1. How many cups of **soft drink, cordial**, or **sports drink**, such as lemonade or Gatorade, do you usually drink in a day? (One cup = 250ml. 1 can of soft drink = 1 ½ cups. 1 x 500ml bottle of Gatorade = 2 cups)

______cups per day

______cups per week

______cups per month

- Doesn’t drink soft drink, cordial or sports drinks
- Don’t know [*DO NOT READ*]
- Refused [*DO NOT READ*]

**[Knowledge of breastfeeding]**

The following questions are about breastfeeding and feeding your child.

The term **exclusively breastfeed** means: The infant receives only breast milk from his / her mother or a wet nurse, or expressed breast milk and no other liquids or solids with the exception of drops or syrups consisting of vitamins, mineral supplements or medicines

1. From what you’ve heard and read, what do you understand to be the recommended age to which you should continue to **exclusively breastfeed** your child?

______months

______years

- Other, please specify ____________________
- Don’t know [*DO NOT READ*]
- Refused [*DO NOT READ*]

1. From what you’ve heard and read, what do you understand to be the recommended age to first give your child solid foods?

______months

______years

- Other, please specify ____________________
- Don’t know [*DO NOT READ*]
- Refused [*DO NOT READ*]

1. On a scale from one to five, how much do you believe that *exclusive* breastfeeding can prevent childhood obesity or overweight issues where 1 is don’t believe at all and 5 is strongly believe?

| don’t believe  at all |  |  |  | strongly believe |
| --- | --- | --- | --- | --- |
| 1 | 2 | 3 | 4 | 5 |

**[Intention to breastfeed]**

1. Do you plan to breastfeed your child?

- Yes
- No 🡪Go to Q19
- Unsure [DO NOT READ] 🡪Go to Q19
- Refused [*DO NOT READ*] 🡪Go to Q19

1. To what age do you plan to **exclusively breastfeed** your child? [Prompt: to what age to you plan to give breastmilk only (by breastfeeding or expressing milk)?

______months

______years

- Other, please specify ___________________
- Don’t know [*DO NOT READ*]
- Refused [*DO NOT READ*]

1. To what age do you plan to breastfeed your child whilst also feeding them other foods e.g. solids or water?

______months ______years

- Other, please specify___________________
- Don’t know [*DO NOT READ*]
- Refused [*DO NOT READ*]

**[Obesity concern]**

1. On a scale from one to five, how concerned are you about your unborn child becoming over weight? [Read scale]

| unconcerned | a little concerned | concerned | fairly concerned | very concerned |
| --- | --- | --- | --- | --- |
| 1 | 2 | 3 | 4 | 5 |

**[Sources of support, trust in source and utility of information]**

Thinking now about where you get information about early childhood parenting, including infant feeding and play, I will read out a list of possible sources of information and ask you which of them you have used.

1. Did you seek or receive information about infant feeding from: [ASK FOR EACH]

| **OPTIONS [Read out]:** | [Only ask follow-up questions (below) if the option YES in the left column is selected]  **[Say] When you got this advice, did you find it** [please select *one* option only]: | |
| --- | --- | --- |
| Family  Yes – ask question to right →  No – go to next option | - Very helpful - Somewhat helpful - Not helpful at all | Are there particular family members? Please describe ___________________  ___________________ |
| Friends  Yes – ask question to right →  No – go to next option | - Very helpful - Somewhat helpful - Not helpful at all |  |
| Child & family nurse  Yes – ask question to right →  No – go to next option | - Very helpful - Somewhat helpful - Not helpful at all |  |
| Other health professional  Yes – ask question to right →  No – go to next option | - Very helpful - Somewhat helpful - Not helpful at all | Please describe (e.g. GP, paediatrician, pharmacist, dietician, lactation consultant)  ___________________ |
| Telephone helpline  Yes – ask question to right →  No – go to next option | - Very helpful - Somewhat helpful - Not helpful at all | Please describe (e.g. Breastfeeding Association, Parent Line NSW, Tresillian)  ___________________ |
| Apps  Yes – ask question to right →  No – go to next option | - Very helpful - Somewhat helpful - Not helpful at all | Could you please tell me which one you use the most?  ___________________ |
| Websites  Yes – ask question to right →  No – go to next option | - Very helpful - Somewhat helpful - Not helpful at all | Could you please tell me which one you use the most?  ___________________ |
| Other  Yes – ask question to right →  No – go to next option | - Very helpful - Somewhat helpful - Not helpful at all | Please provide details ____________________  ____________________  ____________________ |

**[Acculturation scale]**

The following questions are about your language and culture.

[DO NOT READ: Where you see LANGUAGE please substitute your main language other than English (Cantonese, Mandarin, Arabic, other) For example, if you speak mostly Mandarin at home circle the 2 for mostly LANGUAGE]

1. What language do you normally speak at home? (circle one number only)
   1. Only LANGUAGE
   2. Mostly LANGUAGE
   3. English and other LANGUAGE
   4. Mostly English
   5. Only English
2. what language do you normally speak with your friends? (circle one number only)
   1. Only LANGUAGE
   2. Mostly LANGUAGE
   3. English and other LANGUAGE
   4. Mostly English
   5. Only English
3. What language do you prefer? (circle one number only)
4. Only LANGUAGE
5. English and LANGUAGE
6. Mostly English
7. Only English
8. Mostly LANGUAGE
9. What language do you read better? (circle one number only)
10. LANGUAGE
11. English and LANGUAGE equally
12. English
13. What language do you write better? (circle one number only)
14. LANGUAGE
15. English and LANGUAGE equally
16. English
17. What ethnic group do you identify with? (circle one number only)
18. Only another ethnic group
19. Mostly another ethnic group
20. Australian and an other ethnic group equally
21. Mostly Australian
22. Only Australian

b) If another ethnic group, please specify ____________________________

1. In what language do you usually think? (circle one number only)
2. Only LANGUAGE
3. Mostly LANGUAGE
4. English and LANGUAGE
5. Mostly English
6. Only English
7. How important to you is it that the LANGUAGE traditions be honoured/followed (circle one number only)
8. Very important
9. Somewhat important
10. Not very important
11. Not at all important

**[Mother’s diabetes diagnosis]**

1. Have you ever been told by a doctor, nurse or at a hospital that you have diabetes?
2. Yes 🡪 Go to Q31
3. No 🡪 Go to Q32
4. Don't know [DO NOT READ] 🡪 Go to Q32
5. Refused [DO NOT READ] 🡪 Go to Q32
6. What type of diabetes were you told that you had?
7. Type 1
8. Type 2
9. Gestational
10. Other, please specify____________________________
11. Don’t know [*DO NOT READ*]
12. Refused [*DO NOT READ*]
13. Have you ever been told by a doctor, nurse or at a hospital that you have high glucose levels in your blood or urine? [READ LIST]

1 Yes

2 No

3 Borderline

4 Only during pregnancy

5 One time reading / abnormal reading

6 Don't know [DO NOT READ]

7 Refused [DO NOT READ]

**[Demographics]**

This set of questions is about your background such as income, working, and education.

1. In which country were you born?

1 Australia

2 Other, please specify _____________________

3 Refused [DO NOT READ]

1. How many years have you been living in Australia?

______years

- Always lived in Australia
- Don’t know [*DO NOT READ*]
- Refused [*DO NOT READ*]

1. What is your age?

- 16-19
- 20-24
- 25-29
- 30-34
- 35-39
- 40-44
- 45-49
- 50+
- Refused [*DO NOT READ*]

1. What is your present marital status? [DO NOT READ] [PROMPT IF NECESSARY]

1 Married (refers to registered marriages)

2 De-facto partner

3 Divorced

4 Separated but not divorced

5 Widowed

6 Never married

7 Refused [*DO NOT READ*]

1. What is your religion?

- No religion
- Christian
- Islam
- Judaism
- Buddhism
- Other, please specify ____________________________
- Refused [*DO NOT READ*]

1. Before tax is taken out, which of the following best describes your approximate household income range, from all sources? [READ LIST]
2. Less than $10 000
3. $10 000 – $19 999
4. $20 000 – $39 999
5. $40 000 – $59 999
6. $60 000 – $79 999
7. $80 000 – $149 999
8. $150 000 or more
9. Don’t know [DO NOT READ]
10. Refused [DO NOT READ]
11. How would you describe your current employment status? [DO NOT READ] [PROMPT IF NECESSARY]
12. Employed full-time (include self-employed)
13. Employed part-time (include self-employed)
14. Paid maternity leave – employed
15. Unpaid maternity leave – employed
16. Unemployed
17. Home duties
18. Casually employed
19. Student and working
20. Student and not working
21. Retired
22. Full-time carer
23. Unable to work due to health problems
24. Don’t know [*DO NOT READ*]
25. Refused [*DO NOT READ*]
26. What is the highest qualification you have completed?
27. No schooling
28. Not completed primary school
29. Completed primary school
30. Completed years 7 to 9
31. Completed School Certificate or Intermediate Certificate or Year 10 or 4th Form
32. Completed High School Certificate or Leaving Certificate or Year 12 or 6th Form
33. TAFE certificate or diploma
34. University or some other tertiary institute degree or higher
35. Other, please specify _______________________________
36. Don’t know [*DO NOT READ*]
37. Refused [*DO NOT READ*]

**[Survey completion]**

That’s all the questions we have for you PIPE NAME FROM SAMPLE. Thank you for your time with this survey.

Would you like to receive the $20 as:

A Coles gift card or 1

A Woolworths Groceries gift card? 2

PLEASE CONFIRM EMAIL ADDRESS PIPED THROUGH

And what email address would you like us to send that to:

___________________@___________________________ [PIPE EMAIL]

READ BACK SPELLING

IF NO EMAIL ADDRESS, ENTER POSTAL ADDRESS [PIPE ADDRESS]

_________________________________________________

________________________ POST CODE ____________

Thank you for participating in this survey, Again I am ______, from the Healthy Beginnings study conducted by NSW Health.

[IF HAVEN’T HAD BABY YET] All the best with the birth of your child.

We will be in contact again for a follow up survey when your baby is 6 months old.

We will be calling from this same number, so if you want to save that, you will know who it is from.

**End of the survey**

**Thank you very much for taking part in this interview**

Health Promotion Unit

South Eastern Sydney and Sydney Local Health Districts

Level 9 (North) King George V Building

Missenden Road

Camperdown NSW 2050

Phone: 95159055

**Communicating Healthy Beginnings Advice by Telephone (CHAT): for Arabic or Chinese speaking Mothers**

**Mothers 6-month Telephone Survey**

***Please read this section to participant*:**

Good morning/afternoon ____________[mothers first name].

My name is ______, calling on behalf of the Healthy Beginnings program which you registered to participate when you were pregnant. We spoke with you six months ago and did a similar survey.

You have been sent a letter recently advising you that someone from the study group would be calling to talk to you about your experiences of the first 6 months of motherhood.

As a part of the Healthy Beginning study, you should have received some mailed information and also SMS with telephone calls from our nurses to support you. Today, we would like to ask you about that support and some other questions that I’ll explain as we go.

After we complete the survey we will arrange to send you a Coles gift card to the value of $20. This may take a few days to get to you.

As with the previous telephone survey you did with us, all the information collected is confidential. You have a unique identification number and your name and contact details are not on the survey. Your personal details will be kept secure so that only some project staff and I will be able to link your survey answers to you.

**[Survey introduction]**

S1 The questions should take about 30 minutes to complete. Is now a convenient time for us to go through them? It would be helpful if you could be in a quiet place to talk.

**[DO NOT READ RESPONSE OPTIONS]**

- Yes 🡪 SKIP TO S3
- Yes, but not now 🡪 ARRANGE CALLBACK

When is a good day/time for me to ring back?

___________day _________am / pm

- No, I would like to withdraw🡪 CONTINUE TO S2

S2 No problem, is there any particular reason why you would like to withdraw from the study?

**[DO NOT READ RESPONSE OPTIONS]**

1. Yes, please specify________________________________________________
2. Prefer not to say

[**END SURVEY**]

S3 Before we get started, if you don’t feel comfortable answering any question we can move on to the next one, this will not affect your eligibility for your gift voucher. Do you have any questions so far? If any questions arise during this interview, we’ll have time to discuss them at the end.

[RECORD RESPONDENT QUESTIONS HERE – DO NOT ANSWER ANY QUESTIONS OTHER THAN THOSE RELATING TO THIS SURVEY THAT YOU ARE APPROVED TO ANSWER.]

[FOR ANY HEALTH-RELATED QUESTIONS SAY] I have made a note of that and the Healthy Beginnings team will contact you to assist.

|  |
| --- |
|  |
|  |

**[Child Demographics]**

I’d like to start by asking about your baby, [child first name]. You may have already provided some of this information to Healthy Beginnings however I would just like to confirm this is correct.

I am going to be asking you questions about [child first name]’s growth that should be recorded in the Blue Book – your child’s Personal Health Record. If you have this, could you please get it now so that we can check a few things? If you don’t have one, please provide your best estimates.

1. a) Can I confirm you had a boy or a girl? [pipe gender]

- boy
- girl

and their name is? [pipe name]______________________

- - Refused [*don’t read*]

b) Can I confirm your baby's date of birth?

- DD/MM/YYYY [PIPE baby_dob]

1. How much did [child first name]’s weigh at birth? [pipe weight]

[Prompt: Please refer to Blue Book, **page 53 (neonatal information)]**

- - ______ Kilos ______ grams
  - ______ pounds _____ ounces
  - Don’t know
  - Refused [don’t read]

1. What was [child first name]'s length at birth? [pipe length]

*[*Prompt: Please refer to Blue Book, **page 53 (neonatal information)]**

- ______ cm
- ______ inches
- Don’t know
- Refused [don’t read]

1. How much did [child first name]’s weigh at 6-8 weeks of age?

[Prompt: Please refer to Blue Book, **page 69]**

- ______ Kilos ______ grams OR
- ______ pounds _____ ounces
- Don’t know
- Refused [don’t read]

1. What was [child first name]'s length at 6-8 weeks of age?

[Prompt: Please refer to Blue Book, **page 69]**

- ______ cm
- ______ inches
- Don’t know
- Refused [don’t read]

1. When was [child first name]’s 6-8 weeks check completed?

[Prompt: Please refer to Blue Book, **page 69]**

- ______Day ______Month _______Year
- It was not completed [*don’t read*]
- Don’t know [*don’t read*]
- Refused [*don’t read*]

1. Currently, how much does [child first name]’s weigh?

[Prompt: Please refer to Blue Book, **page 78]**

- ______ Kilos ______ grams OR
- ______ pounds _____ ounces
- Don’t know
- Refused [don’t read]

1. Currently, what is [child first name]’s length?

[Prompt: Please refer to Blue Book, **page 78**]

- ______ cm OR
- ______ inches
- Don’t know
- Refused [don’t read]

**[Infant feeding]**

The next questions are about feeding your baby. As I mentioned earlier, you may have already provided some of this information to Healthy Beginnings, but I would like to confirm it today. There are no right or wrong answers, just think about your experiences.

1. Has [child first name] ever been breastfed?

Prompt: ‘Ever breastfed’ means ever given breastmilk, even just once. This includes putting the infant to the breast to feed or giving expressed breastmilk.

- Yes 🡪Go to Q10
- No 🡪Go to Q13
- Refused [*don’t read*]

1. A) Are you currently breastfeeding or giving breast milk?

Note: includes giving other foods, drinks as well. Includes giving expressed breast milk

- yes 🡪Go to Q12
- no 🡪Go to Q10B

10B)Including times of weaning, what was the total time that [child first name] was breastfed

Prompt: Weaning is to start baby on food other than breastmilk.

- ________ weeks 🡪Go to Q11
- ________ months 🡪Go to Q11
- Less than one week. 🡪Go to Q11
- Don’t know 🡪Go to Q12
- Refused 🡪Go to Q12

1. What was the main reason for stopping breastfeeding [child first name]?

- Didn’t have enough breast milk 🡪Go to Q13
- Going back to work 🡪Go to Q13
- Specify ___________________________ 🡪Go to Q13
- Don’t know [*don’t read*] 🡪Go to Q13
- Refused [*don’t read*] 🡪Go to Q13

1. Since this time yesterday, has [child’s first name] been breastfed or given breastmilk?

Prompt: Breastfed includes giving expressed breast milk

- Yes
- No
- Refused [*don’t read*]

1. Since this time yesterday, did [child’s first name] receive any of the following?

i. Vitamins, mineral supplements, medicine €Yes €No

ii. Plain water €Yes €No

iii. Sweetened or flavoured water €Yes €No

iv. Fruit juice €Yes €No

v. Tea or infusion €Yes €No

vi. Infant formula €Yes €No

vii. Solid, soft or semi-solid food (such as mashed or

cooked fruit/vegetables/cereals/egg) €Yes €No

viii. Thickener in bottle/cup (e.g Aptamil) €Yes €No

ix. Other *(specify, e.g. rice porridge water or congee)* €Yes€No *_______________*

1. Has [child first name] ever been given infant formula regularly?

Prompt if necessary: regularly means at least once a day.

- Yes 🡪Go to Q15
- No 🡪Go to Q16
- Don’t know [*don’t read*] 🡪Go to Q16
- Refused [Don’t read] 🡪Go to Q16

1. At what age was [child first name] first given infant formula regularly?

Prompt if necessary: regularly means at least once a day. [FIELD TO NOT PERMIT ENTRY GREATER THAN CURRENT AGE OF BABY]

- - ______ weeks
  - ______ months
  - From birth
  - Less than one week old
  - Don’t know
  - Refused

1. Has [child first name] ever been given any food (Solid, soft or semi-solid food such as mashed or cooked fruit/vegetables/cereals/egg)? Prompt: any food, even to taste

- Yes 🡪Go to Q17
- No 🡪Go to Q20
- Don’t know 🡪Go to Q20
- Refused 🡪Go to Q20

1. At what age was [child first name] first given this food (Solid, soft or semi-solid food such as mashed or cooked fruit/vegetables/cereals/egg)? Prompt: first given food, even to taste

[FIELD TO NOT PERMIT ENTRY GREATER THAN CURRENT AGE OF BABY]

- ____________ weeks
- ____________ months
- Don’t know [don’t read]
- Refused [don’t read]

1. A) Has [child first name] ever been given any food regularly (Solid, soft or semi-solid food such as mashed or cooked fruit/vegetables/cereals/egg)? Prompt: regularly means at least once a day.

- Yes 🡪Go to Q18B
- No 🡪Go to Q19
- Don’t know 🡪Go to Q19
- Refused 🡪Go to Q19

B) At what age was [child first name] first given food regularly (Solid, soft or semi-solid food such as mashed or cooked fruit/vegetables/cereals/egg)?

Prompt: regularly means at least once a day. [FIELD TO NOT PERMIT ENTRY GREATER THAN CURRENT AGE OF BABY]

- ___________ weeks
- ___________ months
- Don’t know [don’t read]
- Refused [don’t read]

1. What was the main reason for giving [child first name] solid food? [DO NOT READ LIST]

- Doctor / nurse / other health professional advised me to
- Friend or relative advised me to
- Read leaflets / saw information that advised me to
- Healthy Beginnings program
- Previous experience (with another baby)
- Not producing enough breastmilk
- Baby was not satisfied with milk
- Baby was not gaining enough weight
- Baby was waking up during the night
- Baby able to sit up and hold food in hand
- Baby seemed ready for solids
- Other reason (Please specify) ________________________
- Don’t know [*don’t read*]
- Refused [*don’t read*]

1. Is [child first name] drinking from a cup?

- Yes
- No
  - Don’t know [*don’t read*]
  - Refused [*don’t read*]

**[Breastfeeding environment]**

1. On a scale of 1 to 5 (where 1 is strongly disagree and 5 is strongly agree), please indicate how much you agree or disagree with the following statement. The women around me, such as friends, family or co-workers, breastfed their children
2. Strongly disagree (None of the women around me breastfed)
3. disagree
4. neither disagree or agree (half breastfed, half did not)
5. agree
6. strongly agree (All of the women around me breastfed)
   - Don’t know [*don’t read]*
   - Refused [*don’t read]*

# **[Tummy time]**

The next questions are about tummy time. Tummy time is when your baby is awake and placed on their tummy for playtime while you are watching them. This can be done on the floor, on your chest or your lap and can help strengthen [child first name]’s head, neck and upper body.

1. At what age did [child first name] start spending time on [his/her] tummy when [he/she] was awake? [FIELD TO NOT PERMIT ENTRY GREATER THAN CURRENT AGE OF BABY]

- __________ days
- __________ weeks
- __________ months
- Don’t know [*don’t read*]
- Refused [*don’t read*]

1. How many days each week does [child first name] spend time on their tummy when he/she is awake?

- None at all 🡪Go to Q26
  - _____________ days a week
  - Don’t know [*don’t read*]
  - Refused [*don’t read*]

1. How many times per day do you place [child first name] on his/her tummy when he/she

is awake?

- __________times a day
  - Don’t know [*don’t read*]
  - Refused [*don’t read*]

1. How long does [child first name] stay on his/her tummy when he/she is awake on

average? Note: for each time, not total

- _________ minutes
  - Don’t know [*don’t read*]
  - Refused [*don’t read*]

**[Mother’s Parenting Confidence]** *(Karitane Parenting Confidence Scale 15-item)*

This next section is related to your parenting experiences and has 15 questions each with 4 options. Please tell me answer that comes closest to how you generally feel about each statement. There are no right or wrong answers.

1. I am confident about feeding my baby
2. No, hardly ever
3. No, not very often
4. Yes, some of the time
5. Yes, most of the time
6. Not applicable (my partner feeds the baby) [*Don’t read*]
7. I can settle my baby
8. No, hardly ever
9. No, not very often
10. Yes, some of the time
11. Yes, most of the time
12. I am confident about helping my baby to establish a good sleep routine
13. No, hardly ever
14. No, not very often
15. Yes, some of the time
16. Yes, most of the time
17. I know what to do when my baby cries
18. No, hardly ever
19. No, not very often
20. Yes, some of the time
21. Yes, most of the time
22. I understand what my baby is trying to tell me
23. No, hardly ever
24. No, not very often
25. Yes, some of the time
26. Yes, most of the time
27. I can soothe my baby when he/she is distressed
28. No, hardly ever
29. No, not very often
30. Yes, some of the time
31. Yes, most of the time
32. I am confident about playing with my baby
33. No, hardly ever
34. No, not very often
35. Yes, some of the time
36. Yes, most of the time
37. If my baby has a common cold or slight fever, I am confident about handling this
38. No, hardly ever
39. No, not very often
40. Yes, some of the time
41. Yes, most of the time
42. I feel sure that my partner will be there for me when I need support
43. No, hardly ever
44. No, not very often
45. Yes, some of the time
46. Yes, most of the time
47. Not applicable (I don’t have a partner) [*Don’t read*]
48. I am confident that my baby is doing well
49. No, hardly ever
50. No, not very often
51. Yes, some of the time
52. Yes, most of the time
53. I can make decisions about the care of my baby
54. No, hardly ever
55. No, not very often
56. Yes, some of the time
57. Yes, most of the time
58. Being a mother / father is very stressful for me
59. Yes, most of the time
60. Yes, some of the time
61. No, not very often
62. No, hardly ever
63. I feel I am doing a good job as a mother / father
64. No, hardly ever
65. No, not very often
66. Yes, some of the time
67. Yes, most of the time
68. Other people think I am doing a good job as a mother / father
69. No, hardly ever
70. No, not very often
71. Yes, some of the time
72. Yes, most of the time
73. I feel sure that people will be there for me when I need support
74. No, hardly ever
75. No, not very often
76. Yes, some of the time
77. Yes, most of the time

# [Traditional Confinement Practices]

The next question is about traditional [Chinese/Arabic] confinement or resting after birth.

# **Did you practice traditional [Chinese/Arabic] confinement, or resting at home in the weeks after your baby’s birth?**

# **Yes**

# **No**

**[Mother’s Dietary Behaviours]**

The next few questions are about food. I’m going to read you a list of different foods and drinks. Please tell me how much of each you usually consume per day or per week. [INTERVIEWER NOTE: ENSURE YOU SPECIFY WHAT A SERVE EQUALS IN THE NEXT TWO QUESTIONS.]

1. How many serves of **vegetables** do you usually eat each day? (Include fresh, dried, frozen and tinned vegetables. One serve = ½ cup cooked or 1 cup of salad vegetables)

- ______serves per day
- ______serves per week
- Don’t eat vegetables
- Don’t know [*DO NOT READ*]
- Refused [*DO NOT READ*]

1. How many serves of **fruit** do you usually eat each day? (Include fresh, dried, frozen and tinned fruit. One serve = 1 medium piece or 2 small pieces of fruit or 1 cup of diced pieces)

- ______serves per day
- ______serves per week
- Don’t eat fruit
- Don’t know [*DO NOT READ*]
- Refused [*DO NOT READ*]

1. How often do you have meals or snacks such as burgers, pizza, chicken, or chips from places like McDonalds, Hungry Jacks, Pizza Hut, KFC, Red Rooster or other takeaway food places?

- ______times per week
- ______times per month
- Rarely or never
- Don’t know [*DO NOT READ*]
- Refused [*DO NOT READ*]

1. How many cups of **soft drink, cordial**, or **sports drink**, such as lemonade or Gatorade, do you usually drink in a day? (One cup = 250ml. 1 can of soft drink = 1 ½ cups. 1 x 500ml bottle of Gatorade = 2 cups). This question does not include diet drinks

- ______cups per day
- ______cups per week
- ______cups per month
- Doesn’t drink soft drink, cordial or sports drinks
- Don’t know [*DO NOT READ*]
- Refused [*DO NOT READ*]

**[Overall Program Satisfaction]**

The next questions are about the service / support you received from Healthy Beginnings as a program/service overall (including the information booklets you received in the mail, SMS messages, and phone calls from nurses).

The next few questions will have a scale with 4 options, I will read these to you. Choose the answer that best fits for you, there is no right or wrong.

| 1. How would you rate the quality of Healthy Beginnings service you received? (information booklets, SMS messages, phone calls from nurses) | ⭘  Excellent | ⭘  Good | ⭘  Fair | ⭘  Poor |
| --- | --- | --- | --- | --- |
| 1. Did you get the kind of support and information you wanted? | ⭘  No, definitely not; | ⭘  No, not really | ⭘  Yes, generally | ⭘  Yes, definitely |
| 1. To what extent has our program Healthy Beginnings (information booklets, SMS messages, phone calls from nurses) met your needs? | ⭘  Almost all of my needs have been met; | ⭘  Most of my needs have been met; | ⭘  Only a few of my needs have been met | ⭘  None of my needs have been met |
| 1. If a friend were in need of similar help (another mother), would you recommend our program to her? | ⭘  No, definitely not; | ⭘  No, I don’t think so | ⭘  Yes, I think so | ⭘  Yes, definitely |
| 1. How satisfied are you with the amount of help you received? | ⭘  Quite dissatisfied | ⭘  Indifferent or mildly dissatisfied | ⭘  Mostly satisfied | ⭘  Very satisfied |
| 1. Have the services you received helped you to deal more effectively with your problems? | ⭘  Yes, they helped a great deal; | ⭘  Yes, they helped somewhat | ⭘  No, they really didn't help | ⭘  No, they seemed to make things worse |
| 1. In an overall, general sense, how satisfied are you with the Healthy Beginnings service you received? (information booklets, SMS messages, phone calls from nurses) | ⭘  Very satisfied | ⭘  Mostly satisfied | ⭘  Indifferent or mildly dissatisfied | ⭘  Quite dissatisfied |
| 1. If you were to seek help again, would you come back to the Healthy Beginnings program? | ⭘  No, definitely not; | ⭘  No, I don’t think so | ⭘  Yes, I think so | ⭘  Yes, definitely |

1. Do you have any comments about any part of the Healthy Beginnings program (information, SMS, booklets, nurses calls) that you would like to share? *[Note: if participant made comments from previous questions, clarify and capture this here. Write exact phrases of the participant where possible]*

|  |
| --- |
|  |
|  |

[**Mother’s employment status**]

The next questions are about your work.

1. How would you describe your current employment status? [If more than one, tick the option highest on the list]

- Employed full-time (include self-employed) 🡪 Go to Q58
- Employed part-time (include self-employed) 🡪 Go to Q58
- Paid maternity leave – employed 🡪 Go to Q59
- Unpaid maternity leave – employed 🡪 Go to Q59
- Unemployed 🡪 Go to Q59
- Home duties 🡪 Go to Q59
- Student and working 🡪 Go to Q58
- Student and not working 🡪 Go to Q58
- Retired 🡪 Go to Q60
- Full-time carer 🡪 Go to Q60
- Unable to work due to health problems 🡪 Go to Q60
- Casually employed 🡪 Go to Q58
- Refused [don’t read] 🡪 Go to Q59

1. How many months after the birth of [child first name] did you return to work and/or

study?

- ____________ months 🡪 Go to Q60
  - Don’t know [*don’t read*] 🡪 Go to Q60
  - Refused [*don’t read*] 🡪 Go to Q60

**[Mother receives support]**

The next section is about support you get from others.

1. During a typical week, is [child first name] cared for by someone other than you?

- Yes 🡪Go to Q61
- No 🡪Go to Q63
- Refused [Don’t read] 🡪Go to Q63

1. And who would that other carer mainly be?

- Please specify_________________________________
  - Refused [*don’t read*]

1. Approximately how many hours per week does someone other than you care for [child first name]?
   - ____________hours/ week
   - Don’t know [*don’t read*]
   - Refused [*don’t read*]
2. Have you lived together with one or both of your parents during parts or all of your baby's first 6 months of life?

- Yes
- No
- Refused *[don’t read]*

1. Have you lived together with one or both of your parents-in-law during parts or all of your baby's first 6 months of life?

- Yes
- No
- Refused *[don’t read]*

**[Sources of support]**

The next two questions are related to where you got information on early childhood parenting since [child name] was born.

1. Where did you get information and advice about early childhood parenting, including infant feeding, sleep and play? [select all that apply]

 Healthy Beginnings

 Friends & family

 Child & Family Health nurse (through the NSW Health Service)

 Family Doctor

 Other health professional

 Telephone helpline

 Social media

 Apps

 Websites

 Other. Please specify ___

1. Where did you most frequently get information to make decisions about breast feeding and formula feeding? [select only one option]:

 Healthy Beginnings

 Friends & family

 Child & Family Health nurse (through the NSW Health Service)

 Family Doctor

 Other health professional

 the Child Blue Book

 Telephone helpline

 social media/ blog

 Apps

 Websites

 Other. Please specify ___

**[Participation in further studies]**

1. Would you be interested in describing and sharing your experiences with the Healthy Beginnings Program in more detail with one of the Healthy Beginnings researchers? (Including the program overall, the booklets and information we provided, and how useful it was for you.) This is optional and if you choose to participate you will be compensated for your time.

- Yes 🡪 Go to Q 65
- No 🡪 Go to Q 68

[*don’t read – If participant asks about the value of compensation for their time in participating in further studies, please specify that there will be a $20 voucher to compensate their time]*

1. Thank you for agreeing to participate in a further interview. It will take 30-45 minutes and take place over the phone. The interview will be with [_____ and with an interpreter].

When is a good day and time for the team to ring you for this interview?

___________day _________am / pm

Before the interview please think about your experience with the Healthy Beginnings Program and what you liked and didn’t like (including the information booklets you received in the mail, SMS messages, and phone calls from nurses).

1. For the further interview we will be looking at the Healthy Beginnings booklets (Chinese- red, Arabic- blue, it will be helpful to have these with you. Did you receive the following information booklets/packages that were mailed to you, and do you still have these?

|  | Yes, I have this | No, I do not have this |
| --- | --- | --- |
| 1. Healthy Beginnings booklet 1 (Antenatal) |  |  |
| 1. Healthy Beginnings booklet 2 (birth–2 months) |  |  |
| 1. Healthy Beginnings booklet 3 (2-4 months) |  |  |
| 1. Healthy Beginnings booklet 4 (4-6 months) |  |  |

1. If you need any of the booklets again, how would like these sent to you?

- Email. Confirm email address [pipe email address] ________
- Post. Confirm address [pipe address] ________
- Not applicable. Participant still has all 4 booklets.

**[Survey Completion]**

That is all the questions we have for you [PIPE MOTHER’S NAME]. Thank you for your time with this survey, and the Healthy Beginnings program.

You will receive your $20 voucher as a Coles gift card sent to you in the mail.

Please confirm your correct mailing address: [PIPE ADDRESS]

CHECK SPELLING. IF NO EMAIL ADDRESS, ENTER POSTAL ADDRESS

_________________________________________________

________________________ POST CODE ____________

Thank you for participating in this survey, Again I am ______, from the Healthy Beginnings study conducted by NSW Health

.

**End of the survey**

**Thank you very much for taking part in this survey**

Health Promotion Unit, Sydney Local Health District

Level 9 (North) King George V Building

Missenden Road, Camperdown NSW 2050 , Phone: 95159055
