# Supplementary material for: Feasibility of a culturally adapted early childhood obesity prevention program among migrant mothers in Australia: a mixed methods evaluation
Source: BMC Public Health. 2021 Jun 16;21:1159. doi: 10.1186/s12889-021-11226-5 (PMC8207722; doi:10.1186/s12889-021-11226-5)
Supplement: Supplementary file 3 — Additional file 3. Interview guide for follow-up with participating mothers. English version of the interview guide with mothers who had participated in the program. Interviews were conducted in language and English. Interviews were co-facilitated by English-speaking staff members and with a bi-lingual staff member or one of two professional interpreters. [file 12889_2021_11226_MOESM3_ESM.docx]

## Additional file 3: Interview guide for follow-up with mothers

**Communicating Healthy Beginnings Advice by Telephone (CHAT): for Arabic or Chinese-speaking Mothers**

**Follow-up interviews with mothers– Interview Guide**

**Introduction**

Hi, my name is [insert facilitator name] and I am from Sydney Local Health District, NSW Health and working on the Healthy Beginnings study that you have been part of. Your baby must be 6 months old now, and you’ve just done a survey over the phone with [research staff name]. Thank you for agreeing to participate in this interview and for taking the time with me today.

**Aim of the interview**

You’ve been invited to participate in this interview because you’ve been involved i the Healthy Beginnings Study working with [Chinese/Arabic] communities on how to improve mums and babies’ health. Healthy Beginnings includes the SMS messages, telephone calls from the nurse, and booklets and resources in the mail.

Today I’d like to talk about your experiences in being part of Healthy Beginnings. The information you share today will help the Healthy Beginnings team, and NSW Health to improve the program and the materials for the [Chinese or Arabic] community. And this will help mums like you, other parents and family members in feeding and raising a healthy baby.

**Format of the interview**

I’ll ask a series of questions to guide the discussion, so please feel free to respond with your thoughts and experiences. We should take approximately 30-60 minutes.

Please feel free to tell me what you think. Don’t be shy to ask questions or ask us to explain more if you don’t understand what I’m asking. This is not a test, and there are no wrong answers or no right answers.

Anything you say here is confidential, which means I will not use your name, but just a number, when we write down what you say. What you say here won’t affect your relationship with Healthy Beginnings program staff or with your local health staff.

**Audio Recording**

You have consented to our discussion being audio recorded so that I can make sure I don’t miss anything and can refer back later. You will not be identified in the recording and your name will not be used. Are you still happy for our discussion to be audio recorded?

[If no objections, start recording] For the sake of the recording, I am confirming that everyone has agreed to this discussion being recorded and we will now get started.

| **Overview of points to cover prior to interview** |
| --- |
| - Thank for agreeing to interview (go over aim of interview if needed) - Introduce bi-lingual staff with Sarah / Marianne present - Will be in Mandarin/Arabic, but also interpret to English for colleagues - Duration 30-60 minutes - We will send you a voucher for your time - Confirm ok with audio recording - If you need to stop at any time, that is ok |

**Interview questions**

| 1. There are four Healthy Beginnings booklets in [Arabic/Chinese] that you received in the mail. (*Refer participant to the booklets; Arabic-Blue, Chinese-Red*).   Did you read the booklets?  In your opinion, how useful are these resources?   1. Why? /Why not? 2. Are there specific changes that you would make? Why? 3. Are there any topics that were particularly well done, or not so well done? Why? 4. Are there other ways that you would prefer to receive the information in the booklets? |
| --- |
| 1. This question is about the text messages (SMS) that were sent to your phone from Healthy Beginnings in [Arabic/Chinese]. Did you read the text messages? How useful and/or relevant was the information and support you received from SMS? Why/why not? |
| 1. For participants who received at least one nurse call in Chinese/Arabic. I understand you received calls from [nurse name] in [Chinese/Arabic] – is that correct? Can you tell me how useful and relevant you found the information/support given to you? Why/why not? |
| 1. For participants who received at least one nurse call in English with interpreter and one in Chinese/Arabic. I understand you received calls from [nurse name] in [Chinese/Arabic], but also from and English-speaking nurse with an interpreter is that correct? Can you tell me about your experience with the different nurses?    1. Did you have a preference? Why/why not?    2. What did you think about the English-speaking nurse using interpreter service? |
| 1. Is there anything that you think could be changed or added to improve the Healthy Beginnings program? Particularly to provide help and information to other mothers and families in the [Arabic / Mandarin]-speaking community? |
| 1. In the Healthy Beginnings program, you received information in relation to your health, can you tell me about what advice was the most useful to you? And why? *Prompt: mother’s health such as physical activity/ being active including pelvic floor muscles, nutrition and healthy foods, your emotional health and self care* 2. When was this - was it a particular time/baby age? 3. Was this from the SMS, booklets and/or nurse calls? |
| 1. In the Healthy Beginnings program, you received information about your baby’s health, can you tell me about what advice was the most useful to you? And why? *Prompt:* *baby’s health including tummy time and play, breastfeeding, introduction of solids and sleep.* 2. When was this - was it a particular time/baby age? 3. Was this from the SMS, booklets and/or nurse calls? |
| 1. Was any of the Healthy Beginnings advice or information you received different to what you had heard before? or new to you? *Prompt: e.g. about breastfeeding, first foods, play time, sleep, your health and wellbeing* 2. Is this different to advice given in your country of birth? How so? 3. Did you follow the Healthy Beginnings advice? Can you comment on why/why not? |
| 1. Can you tell me about your experiences putting baby awake on their tummy (tummy time)? 2. When did you begin and why/what prompted you to start? 3. Did you receive any advice about tummy time? From who? 4. Did the advice from Healthy Beginnings influence you doing tummy time with baby? |
| 1. What would you tell your friends to get them involved in Healthy Beginnings? *Prompt: What do you think is most appealing about this program? What might encourage other mothers to join?* |
| If there is time:   1. To what extent did the Healthy Beginnings program meet your expectations and/or needs?   *Prompt:* *was it what you expected? Why/why not?* |
| If there is time:   1. Did you share the Healthy Beginnings information (the SMS messages, the mailed booklets & the nurse calls), with someone else in your family? Who? Why/why not? 2. If yes, which information / booklets did you share? How useful was this information for other family members? 3. Were there some topics that were new or different for them? 4. Were there some topics they agreed/ disagreed with? |
| 1. We’ve come to the end of all my planned questions. Are there some things we haven’t covered that you would like to add? |
