# Supplementary material for: Feasibility of a culturally adapted early childhood obesity prevention program among migrant mothers in Australia: a mixed methods evaluation
Source: BMC Public Health. 2021 Jun 16;21:1159. doi: 10.1186/s12889-021-11226-5 (PMC8207722; doi:10.1186/s12889-021-11226-5)
Supplement: Supplementary file 4 — Additional file 4. Brief survey and interview guide for follow-up with staff. Brief survey and interview guide with staff who implemented the program. Interviews were conducted in English. [file 12889_2021_11226_MOESM4_ESM.docx]

## Additional file 4: Brief survey and interview guide for follow-up with staff

**Communicating Healthy Beginnings Advice by Telephone (CHAT): for Arabic or Chinese-speaking Mothers**

**Follow-up interviews with Staff – Demographic Survey**

These questions are about your work, work history, and language. You will not be identified (we do not ask your name here) and your answers will be kept confidential.

1. What are your current roles? *(Please specify)*
2. How long have you worked in these roles? *(specify approximate months or years)*
   1. months OR years
   2. months OR years
   3. months OR years

| 1. Do you have prior experience in Australia working with the following client groups? |  |
| --- | --- |
| *(tick all that apply. If ticked, please answer questions in next two columns)* | Months/years of experience working with this group? |
| Mandarin-speaking mothers of young children (0-5 years) | months  years |
| Arabic-speaking mothers of young children (0-5 years) | months  years |

1. What languages do you speak fluently?

| *(Please list the language(s))* | Do you speak this language at home? *(Please tick)* |
| --- | --- |
|  | € only € mostly € sometimes € rarely |
|  | € only € mostly € sometimes € rarely |
|  | € only € mostly € sometimes € rarely |
|  | € only € mostly € sometimes € rarely |
|  | € only € mostly € sometimes € rarely |

1. In which country were you born? *(tick one box)*

- Australia
- Other, please specify _____________________

1. How many years have you been living in Australia? *(write number of years)*

______years, OR € Always lived in Australia

1. a) What ethnic group do you identify with? (*tick one box only*)

- Only another ethnic group, please specify __________________________
- Mostly another ethnic group, please specify __________________________
- Australian and another ethnic group equally, please specify _______________
- Mostly Australian
- Only Australian
- Prefer not to answer

1. What is your religion? (*tick one box only*)

- No religion
- Christian
- Islam
- Judaism
- Buddhism
- Other, please specify ____________________________
- Prefer not to answer

**Communicating Healthy Beginnings Advice by Telephone (CHAT): for Arabic or Chinese-speaking Mothers**

**Follow-up interviews with Staff – Interview Guide**

***Introduction***

Thank you again for agreeing to take part in this interview. Do you have any questions before I start?

My name is [insert facilitator name(s)], from Sydney Local Health District, NSW Health and working on the adaptation of Healthy Beginnings for Chinese and Arabic speaking mothers.

***Aim of the interview***

The interview today will assist us with the evaluation of the cultural adaptation of the Health Beginnings (CHAT CALD Project). The information you provide will help us evaluate the project and give us direction for future work.

As you would be aware, Healthy Beginnings is a program to prevent childhood obesity, supporting mothers with healthy infant feeding and playtime practices from 0-6 months of age. This small trial/feasibility study aimed to culturally adapt Healthy Beginnings to make it relevant to different cultural groups, so that their children and families can also access and gain benefit from the program. For this project, we adapted the program to two language groups: Arabic and Chinese Mandarin.

The research is funded by NSW Ministry of Health, in partnership with the Sydney, South Eastern Sydney, South Western Sydney Local Health Districts. You can find out more online [www.healthybeginnings.net.au/](http://www.healthybeginnings.net.au/).

***Format of the interview***

We will go through a series of questions to ask you about your views on the Healthy Beginnings CHAT CALD project. We should take approximately 30-60 minutes. I’ll be taking a few notes as we talk. Please feel free to ask questions at any time throughout. There are no right, or wrong answers and your answers will be kept confidential, so I encourage you to answer freely. Please have the Healthy Beginnings culturally adapted booklets (4 Chinese, 4 Arabic) nearby for reference.

***Audio recording***

We talked about audio recording in the Participant Information Sheet, but just to say again, I would like to record the interview today. The recording is to ensure that we have an accurate record of everything and we can refer back later. You will not be identified in the recording. If you don’t feel comfortable with having the discussion recorded, then please let me know now.

[*If no objections, start recording*] For the sake of the recording, I am confirming that consent has been obtained to record this interview and we will now get started. Are there any questions before we proceed?

**Interview questions:**

1. In your opinion, how relevant is a culturally adapted Healthy Beginnings program for Chinese and Arabic communities in Sydney?
2. The first four Healthy Beginnings booklets were adapted for Arabic and Chinese mothers, to support them in a culturally- and linguistically-appropriate way, to access and benefit from the Healthy Beginnings program. (*Refer participant to the booklets; Arabic-Blue, Chinese-Red*). In your opinion, how useful are these resources for supporting Chinese and Arabic mothers? Why? /Why not?

| ***Questions for research nurses only***   1. Could you tell me about your experience delivering the Healthy Beginnings advice over the phone with Arabic/Chinese mothers? 2. There were key messages of the Healthy Beginnings program to improve mother’s health (physical activity including pelvic floor, nutrition and emotional wellbeing). What were the best ways you found to talk about these topics? What was most challenging to describe and talk about with [Arabic/Chinese] mothers? 3. There were key messages of the Healthy Beginnings program to improve baby’s health (tummy time and play, breastfeeding, introduction of solids and sleep). What were the best ways you found to talk about these topics? What was most challenging to describe and talk about with [Arabic/Chinese] mothers? 4. You were delivering the phone intervention calls in [Arabic, Mandarin, English with interpreter]. How did you find delivering the advice to [Arabic/Chinese] mothers?    1. How do you feel that your language and cultural background impacted the phone intervention/TINGLE calls with [Arabic/Chinese] mothers?    2. Thinking of the different language and cultural groups within the [Arabic/Chinese] mothers (e.g. from mainland China vs Hong Kong or Egypt vs Syria), how do you feel that your language and cultural background impacted the calls among different groups? 5. When delivering the phone intervention calls there was a script to follow for each of the four calls.   Did you use the scripts? How did you find the content and use the scripts? |
| --- |
| ***Questions for research staff (not nurses)***   1. In your opinion, thinking of the project (the adaptation and implementation of Healthy Beginnings for Arabic and Mandarin- speaking mothers), a) what worked well and b) what presented a challenge(s)? 2. Are there changes that you would suggest to make the Healthy Beginnings program or advice more culturally appropriate and acceptable? What are these changes? 3. In your opinion, how useful was the bilingual nurse role for delivering Healthy Beginnings advice over the phone with Chinese and Arabic mothers? Why/ why not? 4. If the culturally adapted Healthy Beginnings was to continue as a service (e.g. through Local Health District Early Childhood Health Centres), in your opinion, what needs to be considered? 5. If Healthy Beginnings could expand for different cultural groups, are there cultural or language groups that you would suggest/advocate for? Which groups and why? |

1. If the Chinese and Arabic Healthy Beginnings programs were to roll-out as an ongoing service (e.g. through Local Health District Early Childhood Health Centres), in your opinion, what are the best ways to encourage mothers to join?
2. We’ve come to the end of all my planned questions. Are there some things we haven’t covered that you would like to add?

*Thank you for completing this interview.*
