# Supplementary material for: Feasibility of a culturally adapted early childhood obesity prevention program among migrant mothers in Australia: a mixed methods evaluation
Source: BMC Public Health. 2021 Jun 16;21:1159. doi: 10.1186/s12889-021-11226-5 (PMC8207722; doi:10.1186/s12889-021-11226-5)
Supplement: Supplementary file 5 — Additional file 5. Mothers’ satisfaction scores. Participant satisfaction with overall program experience using the Client Satisfaction Questionnaire (CSQ-8) measured at the 6-month survey. Bivariate analyses to assess association between potential explanatory factors and the satisfaction score. [file 12889_2021_11226_MOESM5_ESM.docx]

## Additional file 5: Mothers’ satisfaction scores

**Methods**

Participant satisfaction with the overall experience of the program was measured at the 6-month survey using the validated eight-item Client Satisfaction Questionnaire (CSQ-8)[1], previously translated into Arabic and Simplified Chinese [2] and use assessing client satisfaction with health services. The items used a 4-point Likert scale (see Table 1) to assess perceived satisfaction with various aspects of the program. We calculated the total score by recoding four reverse items and summing the eight items. Our use of the scale was with written permission from the copyright holder (Attkisson) and payment of use fees (2018).

Using IBM SPSS Statistics, descriptive statistics were used to report satisfaction scores. Bivariate analyses were conducted to explore the association between potential explanatory factors and CSQ-8 satisfaction score. Statistical significance was set at p<0.05.

**Results**

Table 1 presents mothers’ self-reported satisfaction with participating in Healthy Beginnings using CSQ-8. Of 129 mothers with complete scores (no missing responses for eight items), the mean score was 28 (± 3.2, range 18-32). Mother’s self-rated satisfaction with the program was higher among the Chinese mothers (mean score 28 ± 2.9, range 21-32) than Arabic mothers (mean score 27 ± 3.4, range 18-32).

To explore differences in CSQ-8 scores, bivariate analyses (linear regressions) were conducted to assess the association with potential explanatory factors (Table 2). When analysing the total sample, language group (Chinese) and first-time mother were correlated with higher satisfaction scores. When analysed by language group, no factors were statistically significant for the Arabic group. For the Chinese group, completing the first nurse call (antenatal) and completing more nurse calls were significantly correlated with higher satisfaction scores.

**References**

1. Attkisson CC, Zwick R. The client satisfaction questionnaire. Psychometric properties and correlations with service utilization and psychotherapy outcome. Eval Program Plann. 1982;5(3):233-7. doi: 10.1016/0149-7189(82)90074-x. PMID: 10259963.

2. Tamalpais Matrix Systems L. CSQ Scales: enhancing client satisfaction advancing services research: CSQ Langauges 2020. [accessed 14 February 2021] Available from: <https://csqscales.com/csq-languages/>

**Table 1.** Mothers’ self-reported satisfaction with the program using the CSQ8

|  | **Total n=135** | | **Arabic n=70** | | **Chinese n=65** | |
| --- | --- | --- | --- | --- | --- | --- |
|  | **n** | **%** | **n** | **%** | **n** | **%** |
| **Client satisfaction questionnaire (CSQ) 8 items** |  |  |  |  |  |  |
| **How would you rate the quality of the service you received?** |  |  |  |  |  |  |
| Poor | 0 | 0.0 | 0 | 0 | 0 | 0 |
| Fair | 4 | 2.5 | 2 | 2.9 | 2 | 3.1 |
| Good | 29 | 17.8 | 15 | 21.4 | 14 | 21.5 |
| Excellent | 102 | 62.6 | 53 | 75.7 | 49 | 75.4 |
| **Did you get the kind of support and information you wanted?** |  |  |  |  |  |  |
| No, definitely not | 5 | 3.1 | 5 | 7.1 | 0 | 0 |
| No, not really | 7 | 4.3 | 5 | 7.1 | 2 | 3.1 |
| Yes, generally | 50 | 30.7 | 21 | 30 | 29 | 44.6 |
| Yes, most definitely | 71 | 43.6 | 37 | 52.9 | 34 | 52.3 |
| **To what extent has the service met your needs?** |  |  |  |  |  |  |
| None of my need have been met | 0 | 0.0 | 0 | 0 | 0 | 0 |
| Only a few of my needs have been met | 9 | 5.5 | 5 | 7.1 | 4 | 6.2 |
| Most of my needs have been met | 87 | 53.4 | 41 | 58.6 | 46 | 70.8 |
| Almost all of my needs have been met | 39 | 23.9 | 24 | 34.3 | 15 | 23.1 |
| **If a friend were in need of similar help would you recommend this service?** |  |  |  |  |  |  |
| No, definitely not | 5 | 3.1 | 4 | 5.7 | 1 | 1.5 |
| No, not really | 2 | 1.2 | 2 | 2.9 | 0 | 0 |
| Yes, generally | 15 | 9.2 | 2 | 2.9 | 13 | 20 |
| Yes, most definitely | 113 | 69.3 | 62 | 88.6 | 51 | 78.5 |
| **How satisfied are you with the amount of help you received?** |  |  |  |  |  |  |
| Quite dissatisfied | 24 | 14.7 | 24 | 34.3 | 0 | 0 |
| Indifferent or mildly dissatisfied | 9 | 5.5 | 5 | 7.1 | 4 | 6.2 |
| Mostly satisfied | 34 | 20.9 | 13 | 18.6 | 21 | 32.3 |
| Very satisfied | 65 | 39.9 | 25 | 35.7 | 40 | 61.5 |
| **Have the services helped you to deal more effectively with your problems?** |  |  |  |  |  |  |
| No, they didn’t help at all | 0 | 0.0 | 0 | 0 | 0 | 0 |
| No, they didn’t really help | 3 | 1.8 | 3 | 4.3 | 0 | 0 |
| Yes, they helped somewhat | 44 | 27.0 | 24 | 34.3 | 20 | 30.8 |
| Yes, they helped a great deal | 86 | 52.8 | 41 | 58.6 | 45 | 69.2 |
| **Overall how satisfied are you with the service you received?** |  |  |  |  |  |  |
| Quite dissatisfied | 2 | 1.2 | 1 | 1.4 | 1 | 1.5 |
| Indifferent, mildly satisfied | 4 | 2.5 | 3 | 4.3 | 1 | 1.5 |
| Mostly satisfied | 33 | 20.2 | 16 | 22.9 | 17 | 26.2 |
| Very satisfied | 95 | 58.3 | 49 | 70 | 46 | 70.8 |
| **If you were to seek help again, would you come back to the service?** |  |  |  |  |  |  |
| No, definitely not | 9 | 5.5 | 9 | 12.9 | 0 | 0 |
| No, I don’t think so | 4 | 2.5 | 2 | 2.9 | 2 | 3.1 |
| Yes, I think so | 19 | 11.7 | 5 | 7.1 | 14 | 21.5 |
| Yes, most definitely | 102 | 62.6 | 53 | 75.7 | 49 | 75.4 |
| **CSQ8 score** [min 8, max 32] |  |  |  |  |  |  |
| Overall score - Mean (SD) * | 28(3.2) |  | 27(3.4) |  | 28(2.9) |  |
| Range | 18-32 |  | 18-32 |  | 21-32 |  |

Note: Values do not equal 100% due to missing values.

*For complete scores with no missing values (n=129 total, n=64 Arabic, and n=65 Chinese)

**Table 2.** Bivariate analyses to assess association between potential explanatory factors and CSQ-8 satisfaction score (for complete scores)

|  | **Total n=129** | | Arabic n=64 | | Chinese n=65 | |
| --- | --- | --- | --- | --- | --- | --- |
|  | B | **P-value** | **B** | **P-value** | **B** | **P-value** |
|  |  |  |  |  |  |  |
| **Language group** (2 categories) | 1.410 | **0.011*** |  |  |  |  |
|  |  |  |  |  |  |  |
| **Years in Australia** (continuous)^ | -0.074 | 0.437 | -0.104 | 0.473 | -0.050 | 0.697 |
|  |  |  |  |  |  |  |
| **First-time mother** (N=0, Y=1) | 1.157 | **0.043*** | .110 | 0.914 | 1.146 | 0.128 |
|  |  |  |  |  |  |  |
| **Number of nurse calls completed**  (5 categories) | 0.309 | 0.149 | -.353 | 0.306 | .605 | **0.048*** |
|  |  |  |  |  |  |  |
| **Early contact – nurse call one (antenatal) completed** (N=0, Y=1) | 1.038 | 0.066 | .590 | 0.496 | 1.717 | **0.015*** |
|  |  |  |  |  |  |  |
| **Education** (3 categories – high school, technical college, university) | 0.213 | 0.493 | -.552 | 0.266 | .128 | 0.813 |
|  |  |  |  |  |  |  |
| **Income** (3 categories – do not know, under AUD$80,000, 80,000+) | -.659 | 0.082 | -.246 | 0.682 | -.237 | 0.628 |
|  |  |  |  |  |  |  |

^Continuous independent - Bivariate correlation, Pearson’s Coefficient.

*p < 0.05
