# Supplementary material for: Feasibility of a culturally adapted early childhood obesity prevention program among migrant mothers in Australia: a mixed methods evaluation
Source: BMC Public Health. 2021 Jun 16;21:1159. doi: 10.1186/s12889-021-11226-5 (PMC8207722; doi:10.1186/s12889-021-11226-5)
Supplement: Supplementary file 6 — Additional file 6. Demographic characteristics of mothers involved in interviews. Demographic characteristics of mothers who agreed to be contacted for an interview at program completion, and those who competed a follow-up interview. [file 12889_2021_11226_MOESM6_ESM.docx]

## Additional file 6: Demographic characteristics of mothers involved in interviews

**Table 1.** Demographic characteristics of mothers who agreed to be contacted for an interview at program completion, and those who competed a follow-up interview

|  | **Arabic-speaking mothers** | |  | **Chinese-speaking mothers** | |
| --- | --- | --- | --- | --- | --- |
| **Variables** | **Agreed to be contacted for interview (n=52)** | **Completed interview (n=22)** |  | **Agreed to be contacted for interview (n=34)** | **Completed interview (n=20)** |
| **Mothers’ age** |  |  |  |  |  |
| 18-24 | 6 | 2 |  | 2 | 0 |
| 25-34 | 31 | 13 |  | 21 | 14 |
| 35-44 | 15 | 7 |  | 11 | 6 |
| **First-time mother** |  |  |  |  |  |
| Yes | 12 | 4 |  | 11 | 10 |
| No | 37 | 18 |  | 23 | 10 |
| **Years in Australia** |  |  |  |  |  |
| < 6 years | 24 | 9 |  | 16 | 10 |
| ≥ 6 years | 19 | 9 |  | 18 | 10 |
| **Annual household income** |  |  |  |  |  |
| < $ 80,000 AUD | 9 | 5 |  | 8 | 6 |
| ≥ $ 80,000 AUD | 2 | 1 |  | 19 | 10 |
| Do not know/prefer not to answer | 41 | 16 |  | 7 | 4 |
| **Employment status** |  |  |  |  |  |
| Employed (full or part-time/casual) | 5 | 1 |  | 19 | 11 |
| Unemployed, home duties, other | 47 | 21 |  | 15 | 9 |
| **Education level** |  |  |  |  |  |
| Up to secondary school | 30 | 12 |  | 2 | 1 |
| Technical college/diploma | 10 | 5 |  | 4 | 2 |
| University degree or higher | 12 | 5 |  | 28 | 17 |
| **Religion** |  |  |  |  |  |
| No religion | 0 | 0 |  | 28 | 15 |
| Buddhism | 0 | 0 |  | 4 | 3 |
| Christianity | 12 | 8 |  | 2 | 2 |
| Islam | 33 | 11 |  | 0 | 0 |
| Mandaeism | 7 | 3 |  | 0 | 0 |
| **Nurse calls completed** |  |  |  |  |  |
| < 2 calls completed | 31 | 10 |  | 4 | 1 |
| ≥ 2 calls completed | 21 | 12 |  | 30 | 19 |
